# The retaining kdo transferase that synthesizes *Escherichia coli* K13 capsule is deeply divergent from structurally homologous enzymes

**DOI:** 10.1016/j.jbc.2026.111162

**Published:** 2026-01-13

**Authors:** Manitabhai Govind, Mikel Jason Allas, Bo-Shun Huang, Todd L. Lowary, Matthew S. Kimber

**Affiliations:** 1 Department of Molecular and Cellular Biology, University of Guelph, Guelph, Ontario, Canada; 2 Department of Chemistry, University of Alberta, Edmonton, Alberta, Canada; 3 Institute of Biological Chemistry, Academia Sinica, Taipei, Taiwan; 4 Institute of Biochemical Sciences, National Taiwan University, Taipei, Taiwan

**Keywords:** bacterial capsule, double-displacement mechanism, glycosyltransferase, 3-deoxy-D manno-octulosonic acid, protein structure

## Abstract

*3-Deoxy-D-manno-octulosonic acid (*Kdo) is an eight-carbon monosaccharide that, in Gram-negative bacteria, is an essential structural component of both lipopolysaccharide and the polymeric linker in Group 2 and 3 capsules. Kdo is also an important building block of the variable region of various capsular structures, but the responsible enzymes have, to date, not been investigated. Here, we structurally and functionally characterize the protein KrkA from *Escherichia coli* capsular serotype K13, showing that it has both ribosyltransferase and Kdo-transferase activity, consistent with this protein being solely responsible for synthesizing the K13 (and the closely related K20 and K23) capsular polysaccharide repeat. We show that the N-terminal module of this protein is the Kdo-transferase. This module’s x-ray structure (at 2.7 Å resolution) resembles that of two characterized Kdo-transferases: the GT99 module of WbbB, and the GT107 module of KpsC, despite sharing negligible sequence similarity. KrkA is therefore the founding member of a new GT family, GT140. KrkA_GT__140_ is organized into a trimer, where a single residue contributes to a neighboring protomer’s acceptor binding site. Using a D193C mutation, we determined the Kdo adduct and ternary complex structures at 2.0 and 2.2 Å resolution, respectively. These structures, along with site-directed mutagenesis, confirm the critical nature of the nucleophile Asp193 and the general base Glu102 (contributed by a secondary structure element distinct from that in GT99 and GT107), as well as important adduct and acceptor binding residues. *Escherichia coli* isolates collectively encode four distinct clusters of related proteins, suggesting polyphyletic origins for this capsular serotype.

*Escherichia coli* is an opportunistic pathogen that imposes an enormous burden on human health, causing, for example, an estimated 23% of global antimicrobial-resistant (AMR)-related deaths in 2019 (1). An important pathogenicity determinant for *E. coli* and other Gram-negative bacteria is the capsule, an extended surface layer of polysaccharides that protects bacterial cells from antimicrobial peptides, as well as both opsonophagocytosis and complement-mediated killing (2). *E. coli* has over 80 known distinct capsular serotypes, which are divided into four groups based upon the strategy by which they are assembled and exported. Group 1 and Group 4 capsule are assembled using a Wzx/Wzy dependent pathway, and primarily cause intestinal infections, while Group 2 and Group 3 capsules are assembled *via* an ABC-transporter dependent pathway, and primarily cause extraintestinal infections (3). *E. coli* capsular polysaccharides from Group 1, Group 2 and Group 3 are generally anionic, a property that has been shown to confer resistance to cationic antimicrobial peptides (4). This negative charge can be imparted by the incorporation of anionic modifications, most commonly the anionic sugar glucuronic acid, but also by other anionic sugars (*e.g.*, galacturonic acid or sialic acid), or anionic modifications including phosphate ions, pyruvate ketal motifs or amino acids. An additional commonly incorporated anionic monosaccharide is 3-deoxy-D-manno-octulosonic acid (Kdo), an eight-carbon keto-sugar with a C1 carboxylate group. Interestingly, Kdo is incorporated into more than half of Group 2 capsules as either the β-furanose (K74, K95), α-pyranose (K6, K16, K24), or β-pyranose (K12, K13, K14, K15, K19, K20, K23 and K97) form, while being absent in capsules from Groups 1, 3 and 4.

The best-known role of Kdo in Gram-negative bacteria is in the core lipopolysaccharide structure, where the addition of a pair of α-Kdo groups to lipid A by WaaA is required for viability (5). In addition, Group 2 and Group 3 capsule biosynthesis is initiated by the addition of a single β-Kdo group to phosphatidylglycerol by KpsS, followed by a KpsC catalyzed polymerization reaction that extends a poly →4)-β-D-Kdo*p*-(2 → 7)-β-D-Kdo*p*-(2- linker (6). KpsC has two distinct GT107 modules, where the N-terminal module (KpsC_N_) adds the β-(2 → 4)-linked Kdo, while the C-terminal module (KpsC_C_) adds the β-(2 → 7)-linked Kdo. A second well-characterized family of β-Kdo transferases is GT99, exemplified by the N-terminal domain of WbbB (WbbB_GT99_) from *Raoultella terrigena*. This enzyme adds a single β-Kdo residue to the non-reducing terminus of the O-antigen, terminating its elongation and potentiating it for export (7, 8). WbbB_GT99_ and KpsC_N_ are distantly related, and the structures of both shows a highly modified GT-B fold, with the N-terminal Rossmann fold domain reduced in size and displaced and relative to the C-terminal Rossmann domain by an α-helical sub-domain formed by the interdomain linker and an extension to the C-terminus (7, 9). KpsC and WbbB have been shown to use a mechanism so far unique among glycosyltransferases (GTs). All other well-characterized retaining GTs use an S_N_i mechanism, where the acceptor, approaching from the same face of the sugar as the leaving group of the donor, is catalytically activated by the donor phosphate, and is transferred directly to the donor monosaccharide in a single step. WbbB and KpsC, in contrast, use a double-displacement mechanism, where a conserved aspartate residue attacks the donor, forming an enzyme–monosaccharide adduct intermediate with inverted stereochemistry (10, 11). After rearranging into a distinct binding site, this α-Kdo adduct is then resolved in a second half-reaction, where the acceptor, activated by a conserved glutamate residue, attacks the adduct, forming the β-linked product with a net retention of stereochemistry. Interestingly, the 5,7-diacyl pseudaminic acid (Pse) transferase from *Acinetobacter baumannii* capsule biosynthesis was recently discovered to also be a KpsC homolog (12), but it is as yet unclear whether it uses the same mechanism.

The variable region of available capsular biosynthesis operons for *E. coli* strains that express β-Kdo-containing capsules do not contain any obvious homologs of KpsC or WbbB. In particular *E. coli* K13 is a type 2 capsule that contains a core →3)-β-D-Rib*f*-(1 → 7)-β-D- Kdo*p*-(2 repeat, with partial acetylation on the C-4 of the Kdo (Fig. 1, *A* and *B*). Two additional closely-related capsular types share the same polysaccharide core structure, with K23 being acetylated on C5 of ribose instead, and K20 lacking acetylation (13). In this work, we investigate the biochemical and structural basis of the synthesis of the K13 capsule from *E. coli*. We show that the K13 polysaccharide is built by a single protein with both ribosyltransferase and retaining Kdo-transferase activities. Structural and mechanistic characterization of the Kdo-transferase domain shows that this domain is very distantly related to KpsC and WbbB, and similarly forms a covalent adduct to Kdo, consistent with all of these enzymes using a similar double-displacement mechanism.

**Figure 1:**
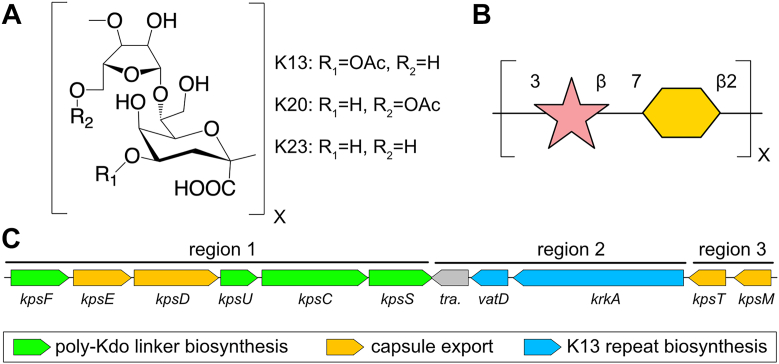
**Details of the K13 capsular antigen and its biosynthesis operon.***A*, chemical structure of the capsular repeat in *Escherichia. coli* capsular serotypes K13, K20 and K23. *B*, the capsular repeat represented in SNFG symbol form. *C*, organization of the capsular gene cluster in *Escherichia. coli* K13 strain NCTC9022.

## Results

### Bioinformatics analysis

**Figure 2:**
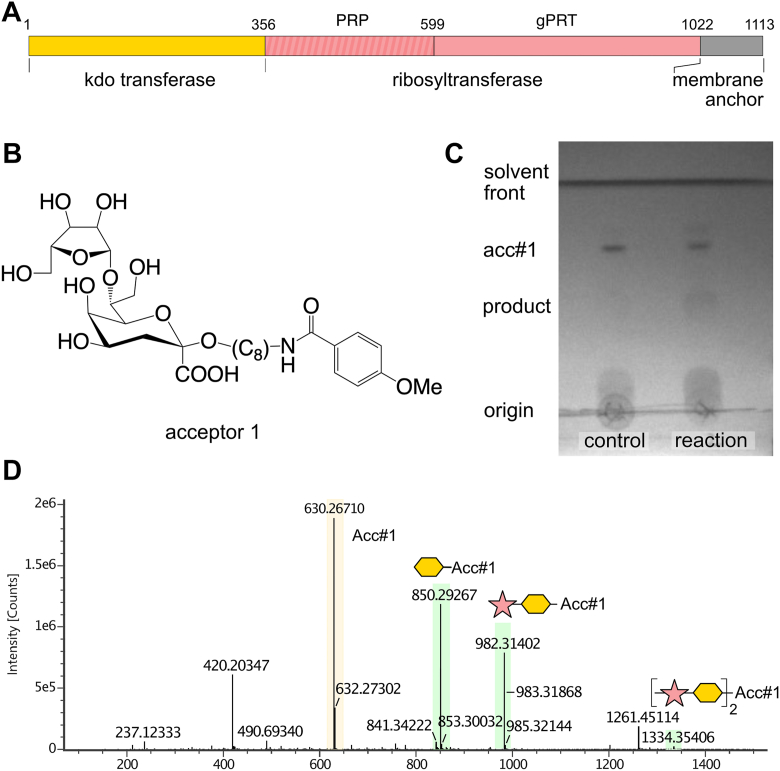
**Reaction catalyzed by full length KrkAΔC.***A*, organization of the KrkA protein; the ribosyltransferase reaction is accomplished by successive action by the glycan acceptor-specific phosphoribosyl transferase and phosphoribosyl phosphatase modules. *B*, the chemical structure of acceptor **1**. *C*, TLC analysis of KrkAΔC reaction containing Kdo, CTP, PRPP, 5-phosphoribosyl-1-pyrophosphate, acceptor 1 (Acc#1), KdsB and KrkAΔC. *D*, the mass spectrum of the KrkAΔC reaction product.

The biosynthesis, assembly, and export of Group 2 capsules is encoded by *kps* gene clusters, which are divided into three distinct regions. Regions 1 and 3 are conserved among serotypes and encode genes that catalyze the synthesis of the common phosphatidyl-glycerol poly-Kdo acceptor, and the proteins necessary to export capsule through the inner membrane, periplasm, and outer membrane (14, 15). Region 2, located between regions 1 and 3, is serotype-specific and encodes for the GTs and any ancillary enzymes (such as sugar nucleotide synthetases) that are required to synthesize the capsular repeat polymer (Fig. 1*C*). However, additional biosynthetic genes can also potentially be encoded elsewhere in the genome (3, 15). In *E. coli* K13 (NCTC9022), region 2 contains three genes (Fig. 1*C*): a transposase; *vatD*, an OatA-related acetyltransferase; and a third large (1113 amino acid) open reading frame (Refseq accession WP_001336789), annotated as a HAD phosphatase, which we here name KrkA. This protein contains distinct N-and C-terminal domains (Fig. 2*A*). The C-terminal domain (residues 357–1013) has a 25% sequence identity (2e^−38^ E-value) to *Klebsiella pneumoniae* O7 O-antigen ribosyltransferase (16), and 29% sequence identity (2e^−31^ E-value) to the ribosyltransferase domain of *Haemophilus influenzae* serotype b capsular biosynthesis protein Bcs3 (17). Polysaccharide ribosyltransferases contain two distinct enzyme modules. The N-terminal module catalyzes the transfer of ribosyl-5′-phosphate from 5-phosphoribosyl-1-pyrophosphate (PRPP) to the non-reducing end of a polysaccharide; this module is designated the glycan acceptor-specific phosphoribosyl transferase. The second module contains a HAD domain phosphatase that subsequently hydrolyses of the 5′-phosphate from the ribose moiety, resulting in the net addition of a ribose group (16, 17); this module is designated the phosphoribosyl phosphatase. In contrast, searching for homologs of the N-terminal domain (residues 1–356) using PaperBlast (18) does not produce any hits, suggesting that homologs of this domain remain experimentally uncharacterized. In particular, this domain shows no significant sequence similarity (BLAST E value > 1) to any previously characterized retaining Kdo (WbbB, KpsC, KpsS) or Pse transferase (KpsS1) (12). Homologs of this domain are commonly found in proteobacteria, with additional examples found in cyanobacteria and firmicutes (Figs. S1, S2).

### KrkA catalyzes the addition of both Kdo and ribose to a synthetic acceptor

A C-terminally His-tagged, full-length construct of KrkA (residues 1–1113) was synthesized and expressed. This construct proved insoluble; examination of an Alphafold 3 model suggested that a C-terminal module containing poorly packed, amphipathic α-helices that are separate from rest of the protein, is likely to mediate membrane association (Fig. S3). This region was therefore removed by deletion mutagenesis. The resulting truncated protein KrkAΔC (residues 1–1022) expressed well and was soluble in the presence of protein stabilizers. The biochemical activity of KrkAΔC was assayed *in vitro* using a synthetic disaccharide acceptor β-D-Rib*f*-(1 → 7)-β-Kdo-2-octyl methoxybenzamide (acceptor **1**; see Fig. 2*B*). This acceptor mimics the growing polymer chain, while the methoxybenzamide aglycone allows detection by UV absorbance. PRPP was used as the ribose donor, as previously established (16); cytosine monophosphate (CMP)-β-Kdo, the Kdo donor, is unstable and was therefore made *in situ* using Kdo, cytosine triphosphate (CTP), and purified CMP-Kdo synthetase (KdsB). A one-pot reaction was performed with both donors and acceptor **1** and product formation was monitored using TLC. TLC showed the gradual disappearance of **1** with the formation of a product which migrated slower on the TLC plate (Fig. 2*C*). Mass spectrometric analysis of this product revealed products of **1** including the addition of a single Kdo residue (*m/z* 850.30), the addition of both a Kdo and a ribose residue (*m/z* 982.31) and the addition of two Kdo plus two ribose residues (*m/z* 1334.35, in low yield) (Fig. 2*D*). This shows that KrkAΔC can iteratively incorporate Kdo and ribose in a manner consistent with this protein being the K13 capsule polymerase, although its relative inefficiency (the major peak remains the unreacted acceptor 1 at *m/z* 630.27), along with its tendency to aggregate, suggests that perhaps only a fraction of the specific construct used in these assays is catalytically active.

**Figure 3:**
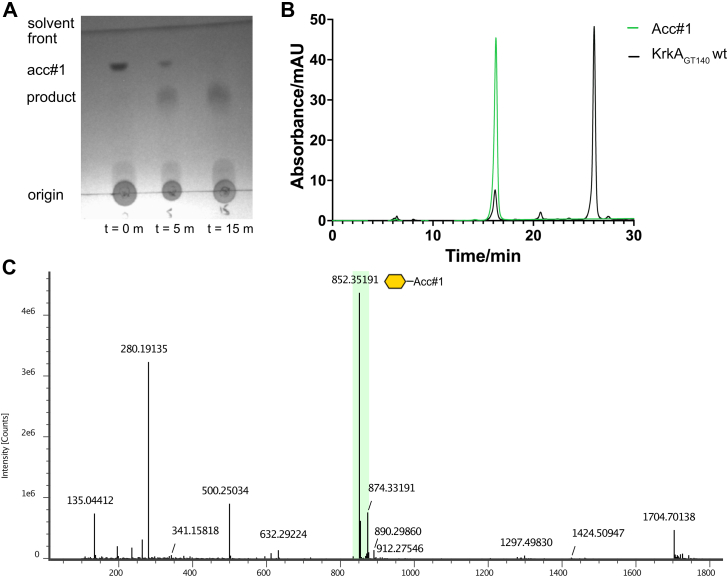
**Reaction catalyzed by KrkA**_**GT140**_**.***A*, TLC analysis of KrkA_GT__140_ reaction containing Kdo, CTP, acceptor 1, KdsB and KrkA_GT__140_. Reaction reached completion after 15 min. *B*, HPLC trace for the reaction of KrkA_GT__140_ (*black*) and acceptor 1 (*green*) showing that the product formed eluted at around 26 min. *C*, mass spectrum of the KrkA_GT__140_ reaction showing the single addition of Kdo as the major peak (*green highlight*).

To investigate the activities of the N-terminal domain of KrkA, a construct termed KrkA_GT__140_, encompassing residues 1 to 356 with a C-terminal His-tag, was synthesized. This construct proved to be highly soluble, and differential scanning fluorimetry confirmed that it is stable (melting temperature = 47 °C) and well-folded; differential scanning fluorimetry also showed a 3 °C stabilization in the presence of 2 mM CMP (Fig. S4), consistent with specificity for CMP-β-Kdo. The activity of KrkA_GT__140_ was assayed using acceptor **1** and *in situ* generated CMP-β-Kdo, with product formation assayed using TLC and HPLC. TLC showed the gradual disappearance of **1** with the formation of a product (Fig. 3*A*). HPLC showed a similar trend, where after the reaction only a trace of **1** (eluting at 16 min) remained, while a new peak appeared (at 27 min). LC-MS using a reverse-phase C18 column was used to analyze this product, corresponding to a major peak eluted at 12.14 min with a [M + H]+ ion peak at *m/z* 852.19 (Fig. 3, *B* and *C*). There is also a small amount of unreacted acceptor at m/z 632.29, and a peak at *m/z* 280.19, which corresponds to fragmentation of the ribose-Kdo disaccharide from the aglycone.

### An Asp193Cys variant is stably modified by kdo

Previous investigations established that the retaining Kdo transferases WbbB_GT99_ and KpsC use a double displacement mechanism (10, 11). A key characteristic of this mechanism is the formation of a covalent glycosyl–enzyme intermediate with the nucleophilic amino acid, which, in both WbbB_GT99_ and KpsC, is an absolutely conserved aspartic acid residue within a QXXXD motif (10, 11). In KrkA_GT__140_, this motif is present as Q_189_TLND_193_, where both Asp193 and Gln189 are absolutely conserved among KrkA_GT__140_ homologs (Fig. S1). The candidate nucleophilic residue, Asp193, was mutated to cysteine, an excellent nucleophile and poor leaving group, and a reasonably good structural mimic of aspartate. In both WbbB_GT99_ and KpsC, an equivalent mutation is stably auto-labelled by endogenous CMP-β-Kdo in the *E. coli* expression host (10, 11). Purified KrkA_GT__140_ D193 C was analyzed using LC-MS, and likewise showed a dominant peak at 43688.75 Da, consistent with a single Kdo residue (220.06 Da) being added to the construct (43466.45 Da) (Fig. S5*A*). This finding suggests that the KrkA_GT__140_ D193C variant, like the analogous WbbB_GT99_ and KpsC constructs, is stoichiometrically modified by the addition of a single Kdo residue. MS analysis of a peptic digest of the product localizes the +220.06 Da modification to the peptide V_187_GQTLNCK_194_, with the fragmentation pattern consistent with Cys193 being the site of Kdo addition (Fig. S5*B*; Table S1). Neither the D193A, D193C nor D193N variants showed any product formation in an HPLC-based assay, suggesting that Asp193 has an indispensable role in Kdo transfer (Table 1; Fig. S6).

**Table 1:** Activity of KrkA_GT__140_ active site variants, assayed by HPLC

Enzyme variant	Enzyme concentration	Product formed
Wild-type	1×	83%
Wild-type	10×	100%
N21A	1×	ND
N21A	10×	4.8%
K71A	1×	ND
K71A	10×	35%
E102A	1×	ND
E102Q	1×	ND
R127A	1×	ND
R127A	10×	ND
R170A	1×	ND
R170A	10×	17%
D193A	1×	ND
D193N	1×	ND
D193N	10×	ND
D193C	1×	ND
H227A	1×	ND
H227A	10×	ND

ND denotes no activity detected. The corresponding traces can be found in Figure S6.

### Structural analysis of KrkA_GT__140_

WT KrkA_GT__140_ failed to crystallize in the absence of ligands; instead, a structure was obtained as a CMP bound complex at 2.7 Å resolutions. Crystals proved to be in the tetragonal space group P4_3_2_1_2, with three molecules per asymmetric unit (ASU). The model encompasses residues 2 to 253, with residues 173 to 174 from the interdomain linker disordered. Data collection and refinements statistics for all structures are shown in Table 2.

**Table 2:** Data collection and refinement statistics

	Wild type	D193C adduct	D193C ternary
PDB ID	9YFX	9YFY	9YFZ
Data collection			
Wavelength (Å)	0.9536	0.9201	0.9201
Resolution range (Å)	46.97–2.7 (2.73–2.7)	40.44–2.027 (2.06–2.03)	41.18–2.214 (2.25–2.21)
Space group	P4_3_2_1_2	P6_3_	P6_3_
Unit cell lengths	132.85 132.85 197.26	121.15 121.15 131.496	121.719,121.719,131.962
Total reflections	3,311,052 (5104)	1,508,683 (73,248)	1,182,869 (59,271)
Unique reflections	131,580 (1094)	70,912 (3520)	55,115 (2735)
Multiplicity	25.2 (4.7)	21.3 (20.8)	21.5 (21.7)
Completeness (%)	99.92 (99.85)	100 (100.00)	100 (100)
Mean I/sigma(I)	6.3 (0.32)	11.1 (1.7)	8.1 (1.5)
Wilson B-factor	82.19	25.66	26.76
R-merge	0.273 (4.499)	0.294 (2.331)	0.457 (3.052)
R-meas	0.2786 (5.014)	0.301 (2.389)	0.468 (3.125)
R-pim	0.05466 (2.114)	0.065 (0.523)	0.101 (0.67)
CC half	0.998 (−0.311)	0.996 (0.367)	0.992 (0.312)
Structure refinement			
R-work	0.2080 (0.3857)	0.2226 (0.3095)	0.2216 (0.3104)
R-free	0.2290 (0.4252)	0.2511 (0.3547)	0.2535 (0.3634)
Protein atoms	8917	5965	5873
Ligand atoms	2	34	84
Solvent molecules	63	256	352
RMS (bonds)	0.003	0.002	0.002
RMS (angles)	0.61	0.55	0.50
Ramachandran favored (%)	95.47	96.81	95.75
Ramachandran allowed (%)	4.15	2.90	3.95
Ramachandran outliers (%)	0.39	0.29	0.29
Rotamer outliers (%)	4.98	0.91	1.23
Clashscore	1.19	0.84	0.51
Average B-factor	100.25	34.69	34.87
Macromolecules	100.44	34.90	35.08
Ligands	85.76	24.48	26.62
Solvent	87.72	32.82	34.27

Statistics for the highest-resolution shell are shown in parentheses.

**Figure 4:**
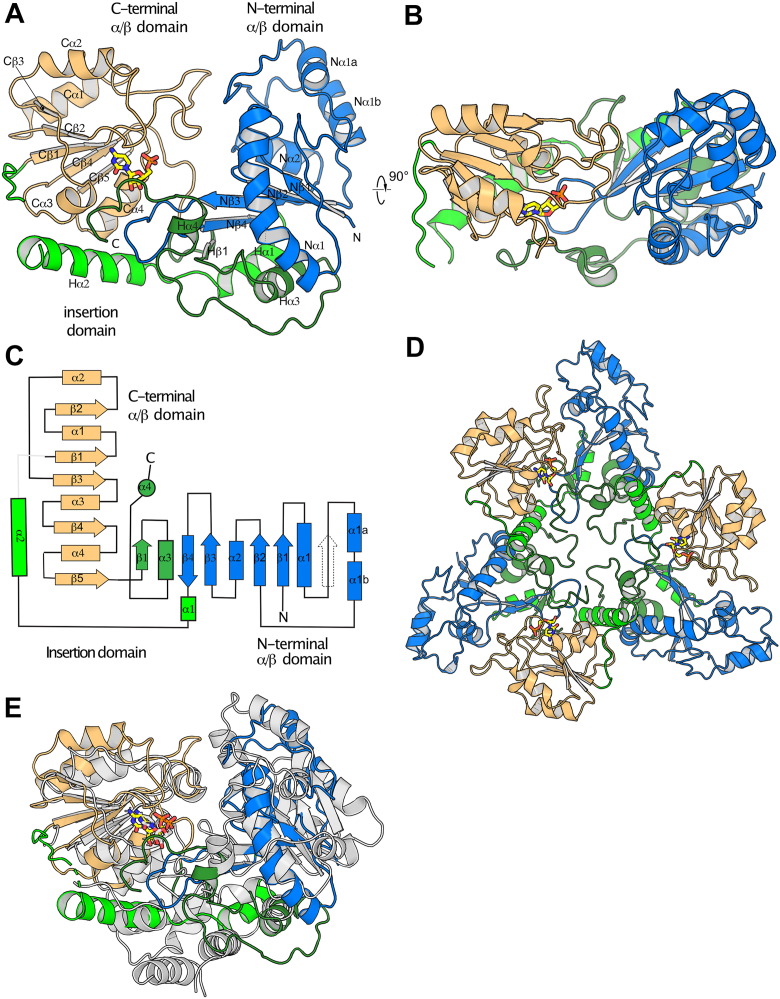
**Overall structure of KrkA_GT__140_.***A* and *B*, orthogonal views of the structure of the protomer, with domains and secondary structure elements as indicated. CMP is shown as yellow sticks. *C*, topology diagram showing the overall organization of the protein. *D,* structure of the trimer. *E*, superposition of WbbB (8mgd, in *white*) on the KrkA_GT__140_ protomer (multicolored).

KrkA_GT__140_ is a GT-B fold glycosyltransferases, with distinct N-terminal and C-terminal Rossmann fold domains. However, these domains are reduced and modified, with additional secondary structure elements contributed by the interdomain linker, and an extension to the C-terminal domain (Fig. 4, *A*–*C*). The C-terminal domain is a relatively unmodified Rossmann fold, containing a five-stranded parallel β-sheet with topology Cβ3, 2, 1, 4, 5, with two relatively short α-helices packed on each face. The N-terminal domain’s β-sheet has mixed topology, with strand order Nβ1, 2, 3, −4, Hβ1; strands Nβ1, 2 and 3 correspond to strands β1, β4 and β5 of a canonical Rossmann fold domain (with topology β3,2,1,4,5,6); the region equivalent to β2 is present as an extended loop that makes three intra-backbone hydrogen bonds with β1, but does not meet PyMol’s criteria for forming a β-strand. Supplementing this core, Nβ4 pairs antiparallel with Nβ3 to form a β-hairpin, where the Nβ3 – Nβ4 loop extends into the active site; and Hβ1 is an additional β-strand contributed by the C-terminal extension. In contrast to WbbB and KpsC, the residues joining the Rossmann domains and extending from the C-terminal Rossmann domain do not form a defined helical domain but are largely integrated into the N-terminal domain, forming additional β-strands and α-helices, while Hα2 packs against the C-terminal domain.

KrkA_GT__140_ is oligomerized into a symmetric trimer, with a single trimer occupying the asymmetric unit (Fig. 4*E*). PISA (19) indicates that the interface buries 1068 Å^2^ per protomer and is predicted to be stable. Most interchain interactions are mediated by the Hα2, which packs against the extended helix α1 in the N-terminal domain and C-terminal extension of a neighboring protomer.

Searching DALI (20) with the KrkA_GT__140_ structure reveals that the closest structural homologs are the retaining Kdo transferases WbbB_GT99_ (Z score = 12.9) and KpsC_N_ (Z = 10.6), but surprisingly, the GT38 polysialyltransferase from *Mannheimia haemolytica* (5wcn) gives a very similar score (Z = 10.2) (Table S2). This score appears to be almost entirely driven by similarity in the C-terminal domain, which produces very similar Z-scores when searched alone, while the N-terminal domain shows only weak structural similarity to any other protein, including other β-Kdo transferases. Of note, WbbB_GT99_ and KpsC_N_ are more similar to each other than they are to KrkA_GT__140_ (Z = 14.8), suggesting that KrkA_GT__140_ is a relative outlier among all β-Kdo transferase-like proteins characterized to date (Fig. 4*D*, Table S3).

### CMP binding site

**Figure 5:**
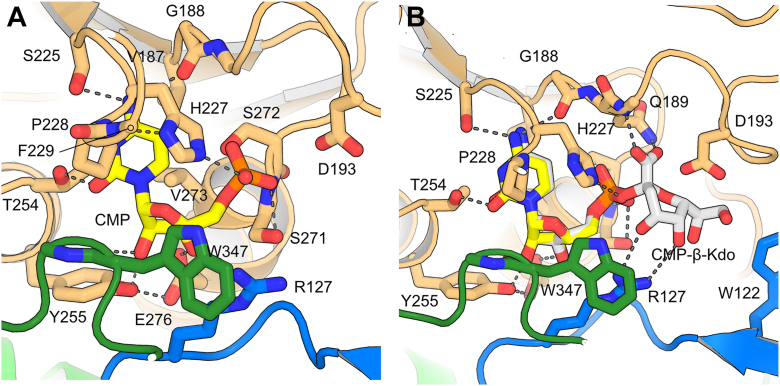
**CMP binding, and model of CMP-β-Kdo binding.***A*, CMP binding in the WT KrkA_GT__140_ binding site. *B*, model of CMP-β-Kdo binding. The structure of WbbB with CMP-β-Kdo bound (8csb) was superimposed on KrkA_GT__140_, and then manually adjusted.

CMP is bound in the active site in all three structures, including the WT protein. In each case, CMP adopts a near identical conformation in all independent protomers, with clear electron density (Figs. 5*A*, S7). CMP interacts primarily with the C-terminal domain, with additional interactions made by both the interdomain linker and the C-terminal extension. The cytosine ring sits in a pocket with Val273 packed on one face, and Pro228 on the other. Pro228 is part of the (K/R)XHP motif conserved among both sialyltransferases (GT families 29, 38, 42, 52, and 80) and retaining Kdo transferases (GT families 99 and 107). The lysine/arginine residue of this motif, which usually hydrogen bonds with O2 and N3 of the cytosine ring through its amine/guanine group, is here replaced by Ser225, which hydrogen bonds with N4 through its hydroxyl group (as does Gly188 through its amide oxygen atom). The cytosine C2 carbonyl is instead hydrogen bonded to Thr254 Oγ (as well as a structural water), while N3 remains unpaired. The ribose moiety is stabilized by hydrogen bonds between the C2 hydroxyl group and both the phenolic oxygen of Tyr255 and the carbonyl oxygen of Gly348; and between the C3 hydroxyl group and the Glu276 carboxylate. The Trp347 and Arg127 side chains form a cation–π pair, which stacks on O1, C4 and C5 of the ribose. Both Trp347 and Gly348 are contributed by the end of the C-terminal extension. The CMP phosphate group makes hydrogen bonds to Ser271Oγ, Ser272N and Oγ, His227Nε2 from the (K/R)XHP motif. It should be pointed out that the His227 imidazole Nδ1 accepts a hydrogen bond from Phe229NH, implying that this atom is deprotonated and that His227 is uncharged and unsuited to act as a proton donor. The H227A variant had no detectable activity, implying that this hydrogen bond is essential for activity (Table 1, Fig. S6). Note that the CMP nucleotide is relatively buried; residues 347 to 351 at the C-terminus have somewhat elevated atomic displacement parameters, consistent with this region undergoing order–disorder transitions to allow donor binding and product release.

### Donor binding model

We could not capture the CMP-β-Kdo donor bound structure, but instead modeled this complex by superimposing the structure of the WbbB_GT99_ donor complex (PDB i.d. 8csb) (10) on KrkA_GT__140_, and then manually adjusting the fit of the nucleotide sugar. This model shows that the active site will readily accommodate the donor, with the C1 carboxylate group forming hydrogen bonds to the amide nitrogen atoms of both the side chain amide nitrogen of Gln189 (of the QXXXD motif), as well as the main chain amide nitrogen of Thr190. The only other potential direct hydrogen bonds are through the Arg127 guanidine, which could potentially hydrogen bond with O4 and/or O5. In this binding mode, positions C7 and C8 are in a relatively non-polar position, packing against Trp122, suggesting that the C7 and C8 hydroxyl groups would be oriented to face the solvent.

### Kdo–Cys193 adduct complex

**Figure 6:**
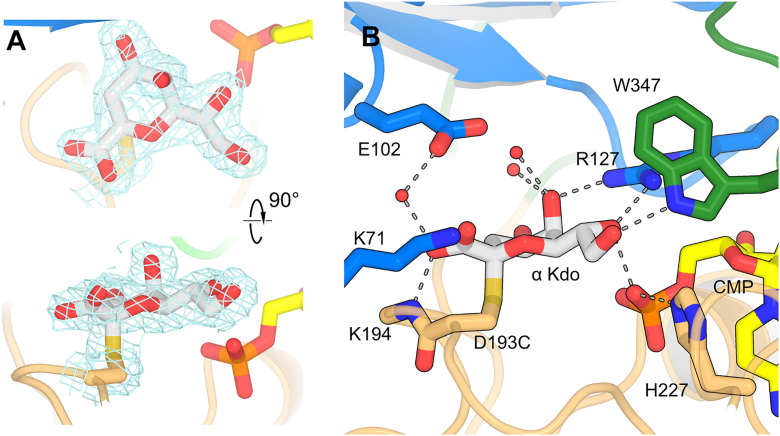
**Details of the α-Kdo-D193C adduct.***A*, orthogonal views of the 2F_o_ – F_c_ electron density map contoured at 1.0 σ around the Kdo group and cysteine residue. *B*, details of the adduct binding site.

Crystals of KrkA_GT__140_ D193C were grown in the presence of 2 mM CMP. These crystals diffracted to 2.03 Å resolutions and proved to be in the hexagonal space group P6_3_. This crystal form has two molecules per ASU, each of which represents an independent trimer, with each oligomer sitting on a crystallographic three-fold axis. For both D193C structures, both tetragonal and hexagonal crystals grew in similar conditions, with the hexagonal datasets yielding slightly higher resolution. As anticipated from the mass spectrometry results, clear electron density is observed for an α-Kdo adduct covalently attached to Cys193, and sitting in a single, well-defined conformation (Fig. 6, *A* and *B*). The α-Kdo ring can be conceptually divided into two hydrophilic ridges separated by O2 and C3, with one ridge having the C2 carboxylate group, and the other characterized by a series of hydroxyl groups extending from C4 to C8. The C1 carboxylate makes a hydrogen bond with a water molecule and Lys194N; the angle is non-ideal, but possibly the slightly greater spacing afforded by the WT Asp193 adduct would allow better geometry. Lys71 is also located close to the C1 carboxylate, and could possibly reorient to be closer; a K71A mutation shows around 4% of WT activity (Table 1, Fig. S6), consistent with this electrostatic interaction playing an important role; however, this residue is not conserved among more remote KrkA homologs (Fig. S1). On the opposite face, O5 and O7 form hydrogen bonds with the guanidine group of Arg127; the C7 hydroxyl group also makes an additional hydrogen bond with CMP phosphate, while the C8 hydroxyl group hydrogen bonds to Trp347 from the C-terminal loop. Additional water-mediated hydrogen bonds are made to O4 and O5.

### Ternary complex

**Figure 7:**
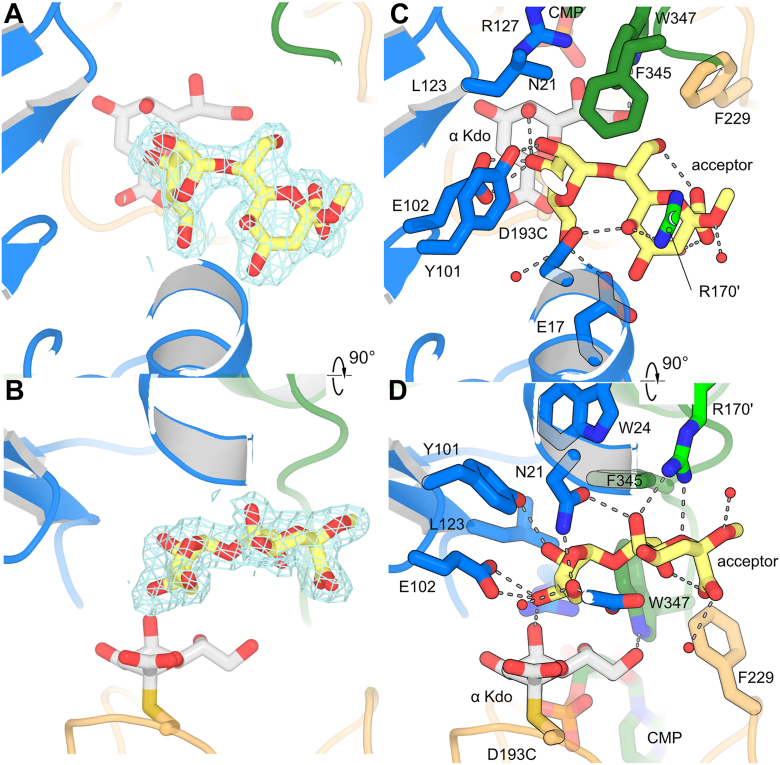
**Acceptor 1 binding in the ternary complex.***A* and *B*, 2F_o_ – F_c_ electron density map contoured around acceptor 1 at 1.0 σ. *C* and *D*, details of interactions of **1** in the active site.

To capture the structure of the ternary complex, crystals of the D193 C variant were grown in the presence of both 2 mM CMP and 2 mM acceptor **1**. These crystals diffracted to 2.2 Å, and were in spacegroup P6_3_, with two molecules per ASU. In neither forming the ternary complex, neither CMP, the α-Kdo adduct nor the protein itself shift significantly from the Cys–α-Kdo adduct structure. The β-D-Rib*f*-(1 → 7)-β-D-Kdo*p* disaccharide binds in a pocket formed where the N-terminal domain, C-terminal domain, interdomain linker and C-terminal extensions all meet (Fig. 7). The ribose C3 hydroxyl group, the attacking hydroxyl group, is hydrogen bonded to Glu102; this residue is therefore proposed to act as the general base. Consistent with this role, the E102A and E102Q variants showed no detectable activity (Table 1; Fig. S6). Interestingly, Glu102 is topologically distinct from the general base in WbbB (Glu158) and KpsC (Glu66), which is instead contributed by the residue equivalent to Trp122 at the C-terminal end of the adjacent β-strand (Fig. S8). Despite their different origins in the structure, the carboxylate groups of these different bases converge upon a very similar location. Ribose O3 also makes an additional hydrogen bond with the axially positioned enzyme-bound α-Kdo O5 hydroxyl group. An equivalent hydrogen bond is also formed between the acceptor and the adduct in both WbbB and KpsC. Ribose O3 is positioned 3.7 Å from the anomeric carbon atom of the donor Kdo adduct (C2), and in line with the C2–Cys193Sγ bond; it is therefore ideally placed for nucleophilic attack. Glu102 and the phenolic hydroxyl group of Tyr101 form hydrogen bonds to ribose O4. The ribose C5 hydroxyl group is hydrogen bonded to both the Glu17 carboxylate and the Asn21 amide nitrogen, as well as to a structured water molecule. The β-Kdo residue of the acceptor makes relatively few hydrogen bonds to the protein, with Arg170 (contributed by an adjacent protomer) making hydrogen bonds to O5 and the endocyclic oxygen through its guanidine side chain, and the Asn21 amide oxygen also hydrogen bonding to the C5 hydroxyl group. Consistent with these residues playing important roles, N21A, R127A and Arg170A mutations show severely reduced turnover, with N21A and R170A showing 0.6% and 2% of WT turnover, respectively, while R127A showed no detectable turnover (Table 1; Fig. S6). The acceptor Kdo group also forms an internal hydrogen bond between the carboxylate oxygen and C8 hydroxyl, as well as hydrogen bonds to structured water molecules. Phe345 and Trp347 stack on both monosaccharides, but the Kdo group is otherwise relatively solvent exposed. This binding mode suggests that the enzyme is highly specific for β-ribofuranose, but possibly has less specificity for the group attached to the ribose anomeric carbon.

The K13, K20 and K23 capsules differ in that K23 is non-acetylated, K20 is partially acetylated on O5 of ribose, while K13 is partially acetylated on O4 of Kdo (Fig. 1, *A* and *B*). In the ternary structure, O5 of ribose and O4 of Kdo approach one another, with Glu17 being close enough to hydrogen bond with both. Kdo O4 faces the bulk solvent, while the ribose O5 sits in a pocket occupied by three water molecules; this suggests that O-acetyl modifications of these groups can be accommodated in the active site with minimal disruption to the existing interactions.

### Alphafold model of full length KrkA

We were unable to crystallize KrkAΔC, but we could generate Alphafold 3 (21) predictions of the trimeric structure of the protein with medium to high pLDDT scores (>70) for the Kdo transferase and ribosyltransferase modules, with lower confidence for interdomain linkers and the C-terminal region (Fig. S9). The model of the Kdo transfer domain agrees well with the experimental structure, with r.m.s.d. values of 1.1 Å. The C-terminal ribosyltransferase module also closely resembles those of known polysaccharide ribosyltransferases, with both the HAD domain (Z-score 25.9, rmsd 1.8 Å) and PRPP-transferase domains (Z-score 23.4, rmsd 4.3 Å) being strongly similar to *H. influenzae* capsular biosynthesis protein Bcs3 (pdb i.d. 8a0m). Interestingly, separately predicted models disagree as to the position of this module (Fig. S9*C*). Alphafold expected position error plots are consistent with the placement of the ribosyltransferase modules relative to other elements of the structure being highly uncertain (Fig. S9*D*), and the model shows minimal contacts between domains. This implies an organization where the N-terminal Kdo transferase domain forms a central hub that tethers the protomers into a trimeric complex, while the ribosyltransferase domains are relatively free to move. This contrasts with other dual domain glycosyltransferases including WbbM and *E. coli* K4 chondroitin synthase, where the fused domains interact strongly and are fixed in position relative to one another (22, 23). We note that searching the structural database with Alphafold 3 models, much like the experimental structure, readily discovers the structural relationship between KrkA, and KpsC and WbbB. These models therefore are broadly useful in identifying potentially informative distant homologs for highly divergent genes found in other bacterial polysaccharide biosynthesis loci.

## Discussion

The capsular polysaccharides produced by *E. coli* K13, K20 and K23 share a common backbone, and only differ in the presence and location of an O-acetyl group. KrkA is absolutely conserved between K13 and K23 strains and the K20 enzyme differs by only three conservative substitutions at sites remote from the active site (Fig. S1). These proteins then share identical active sites that can accommodate acceptors with or without acetylation on either the C4 hydroxyl group of Kdo, or the C5 hydroxyl group of ribose. The differences between these serotypes are likely solely dependent on the presence of the cognate acetyltransferase.

Recent work characterizing the 5,7-diacetyl Pse transferase (KpsS1) from *A. baumannii*, established that this enzyme forms a new GT family, GT118, that is related to WbbB_GT99_ and KpsC, with 30% sequence identity to *E. coli* KpsS (12). This enzyme is a retaining Pse transferase, but, in contrast to retaining Kdo transferases, the donor and product are both in the α-configuration, suggesting that the mode of both donor and adduct binding are likely profoundly different than what is observed in the retaining Kdo-transferases. Interestingly, KpsS1 has Glu132 in a position equivalent of Glu102 in KrkA, with both occurring as a YE motif; because this enzyme lacks the alternative WbbB-equivalent glutamate, this is likely the catalytic base in this enzyme. KrkA and KpsS1 are deeply divergent, while KpsC and WbbB are significantly more closely related. Therefore, the location of the general base in KrkA may be the ancestral position of this group, and the shift to the location seen in WbbB/KpsC a specific adaptation within this lineage.

The availability of a structure of the ternary complex for Kdo transferases from three different GT families allows the identification of conserved and recurring elements within the active site. Key conserved elements include the nucleophilic Asp and Kdo carboxylate binding Gln of the QXXXD motif, and the HP CMP binding motif; these are all located in the C-terminal Rossmann domain (Fig. S10). The Kdo adduct site is flanked by a pair of basic residues. One of these binds the α-Kdo carboxylate and is contributed by a different region of the protein in each case; the other interacts with a ridge of hydroxyl groups located on C4 to C8. Moreover, whereas WbbB and KrkA use a topologically conserved arginine to bind the hydroxyls, KpsC uses a Lys from elsewhere in the structure. Overall, elements contributed by the C-terminal domain module that binds CMP-β-Kdo and initiates adduct formation are highly conserved, whereas those that form the Kdo-adduct binding site and catalyze the second half reaction are more variable. Another notable commonality between KrkA, WbbB_GT99_ and KpsC ternary complexes is that the incoming attacking hydroxyl group of the acceptor is positioned and stabilized by the axially positioned C5 hydroxyl group of the Kdo donor, with this hydroxyl group necessarily acting as a hydrogen bond donor as the general base engages the hydrogen atom on the acceptor hydroxyl group. In the case of Pse, the C5 substituent is an axial acetylated amine, suggesting the potential to donate a hydrogen bond to the reactive hydroxyl group of the acceptor. In contrast, most oct-and non-ulosonic acids, including sialic acids, have an equatorial C5 substituent, which cannot not make an equivalent interaction. This suggests that retaining Kdo-like GTs may not readily evolve specificity for the most common ulosonic acids, including sialic acids.

Phylogenetic analysis suggests that there are five independent clusters of *E. coli* GT140 homologs where each GT140 domain is fused to a ribosyltransferase domain, and with >98% sequence identity to other cluster members (Figs. S1, S2). These additional clusters are exemplified by the sequences E_coli_1 (69% sequence identity to K13 KrkA), E_coli_2 (48% sequence identity), E_ coli_3 (46% identity) and E_coli_O25b (41% sequence identity). These sequences are more distantly related to K13 KrkA than those in other species such as *Yersinia*, suggesting that each of these clusters may have arisen by independent horizontal gene transfer events from different donor species. However, each of these sequences, with the exception of E_coli_O25b, conserves all of the key active site residues with K13 KrkA, including those involved in recognizing the acceptor. This suggests that these 4 *E. coli* groups (as well as numerous other bacteria) synthesize the same polysaccharide backbone (though different patterns of acetylation or other modifications are possible) and that K13/K20/K23 capsules in *E. coli* may have a polyphyletic origin. Interestingly, *E. coli* O16:H5-ST131 strains (E_coli_O25b in Figs. S1 and S2) has 41% sequence identity to KrkA, but shows multiple substitutions in the acceptor binding site. The presence of this protein suggests that GT140 enzymes are involved in the synthesis of at least one additional *E. coli* capsular serotype. No capsular serotype has been documented for this strain, but the presence of both a retaining Kdo transferase and a ribosyltransferase suggests that this protein may synthesize either a K19 or K97 capsule.

*E. coli* capsular serotype K13 is of clinical interest as one of the more prevalent capsular serotypes among extraintestinal pathogenic *E. coli* (ExPEC) strains (24), specifically being a prevalent capsular serotype in urinary tract infections. This capsule has potential as a possible vaccine target, as indicated in a study where the K13 capsule coupled to diphtheria toxin showed protection in mice against renal infection (25). Our findings here help elucidate the biochemical and structural basis for the biosynthesis of this polysaccharide, will assist genetic serotyping efforts, and will help lay the foundation for potential future K13 vaccine glycoengineering efforts.

## Experimental procedures

### Bioinformatics

The NCBI non-redundant database, the Swissprot database and paperBlast were searched using the KrkA (GenBank ID VED05356.1) sequence from *E. coli* K13 (NCTC9022) using the Blastp search algorithm with the default parameters. Structural models for KrkA and KrkA_GT__140_ were prepared using AlphaFold 3.0 (21). The multiple sequence alignment was generated using ClustalOmega (26), and the phylogenetic tree by phylogeny.fr (27). DALI was used to search the Protein Data Bank (PDB) for structurally similar proteins (20).

### DNA methods

*E. coli* KdsB was a generous gift from H. Brade (Research Centre Borstel, Leibnitz Centre for Medicine and Biosciences, Borstel). KrkA_GT__140_-His6 pET-28a and the full length KrkA-His6 pET-28a were synthesized by Twist Bioscience. KrkA_G__T140_ variants were made through site-directed mutagenesis using the Quikchange method and primers (Thermo Fisher Scientific; see Table S4), or else obtained from Twist Bioscience. Amplicons for KrkA_GT__140_ variants were generated through PCR using an in-house recombinant stock of Pfu x7 DNA polymerase according to the Promega Pfu DNA polymerase protocol with KrkA_GT__140_-His6 pET-28a plasmid as template. KrkAΔC-His_6_ amplicon was generated using the Platinum SuperFi II polymerase (Invitrogen) and KrkA-His_6_ plasmid according to manufacturer’s instructions. Both KrkA_GT__140_-His6 and KrkAΔC-His6 pET-28a plasmids were purified according to manufacturer’s instructions using PureLink Quick Plasmid Miniprep Kit (Invitrogen). The parental DNA from the PCR reactions were digested using *Dpn1* (New England Biolabs). The samples were then transformed into chemically competent *E. coli* DH5α, produced in-house through the heat-shock method. The cells are plated on LB agar containing 50 μg/ml of kanamycin which was incubated overnight at 37 °C. Constructs were then sent for Sanger sequencing either at the Advanced Analysis Centre’s Genomics Facility at the University of Guelph or through whole plasmid sequencing at Plasmidsaurus Inc. Once confirmed, small cultures were grown at 37 °C overnight from single isolated colonies in 5 ml of LB broth containing kanamycin (1:1000). Plasmids were then purified using the above-described method and transformed into *E. coli* BL21(DE3), made in-house through the heat shock method. Glycerol stocks were made for both the *E. coli* DH5α and *E. coli* BL21(DE3) cells from the small cultures for later use.

### Protein expression

5 ml starter culture was inoculated with the specific *E. coli* BL21(DE3) cells along with kanamycin and grown overnight at 37 °C. The saturated culture was then transferred to 1 L of 2 YT media containing kanamycin in a 1:1000 ratio and allowed to grow until an OD_600nm_ of 0.6 to 0.8. Dioxane-free IPTG was added to the culture flask to a final concentration of 1 mM and incubated at 16 °C overnight. Cells were harvested through centrifugation at 5000×*g* for 30 min followed by resuspension in 30 ml extraction buffer (50 mM Tris pH 9.0, 250 mM NaCl, 40 mM imidazole, and 5% glycerol) and 1 mM phenylmethylsulfonyl fluoride (Acros Organics). For immobilized metal affinity chromatography purification, a 1 ml HisTrap HP column (Cytiva) was attached to a MasterFlex peristaltic pump and after loading, the protein was eluted using a step-wise gradient (20 mM, 65 mM, 150 mM and 300 mM) of imidazole. Coomassie stained SDS-PAGE gels were used to identify the pure fractions, which were pooled and desalted using gravity columns (Bio-Rad Econo-Pac 10DG) into either a minimal buffer (20 mM Tris-HCl pH 8.0 and 150 mM NaCl) for KrkA_GT__140_ and its variants, or for KrkAΔC the minimal buffer with 10% glycerol. Absorbance at 280 nm was used to measure protein concentrations using extinction coefficients predicted by the ExPASY ProtParam tool. Purified KrkA_GT__140_ produced a band between 37 and 50 kDa on an SDS-PAGE gel with minimal contaminants, consistent with its theoretical molecular weight of 43.48 kDa. Approximately 25 mg of KrkA_GT__140_ was purified per liter of culture. Protein was aliquoted, flash frozen in liquid nitrogen and stored at −80 °C for downstream experiments. Differential scanning fluorimetry was used to confirm that all KrkA_GT__140_ protein variants unfold cooperatively and are stabilized by CMP. Assays were run in triplicate with 5x SYPRO dye added to 1 mg/ml of protein in equal amounts for the apo reaction while 2 mM of CMP was added for the CMP-bound reaction. Melting was monitored from 20 °C to 95 °C on a StepOnePlus Instrument. Data was plotted and analyzed using GraphPad Prism V8.3.

### Reaction assays

Standard reactions (20 μl) for KrkA_GT__140_ were carried out at 37 °C for 30 min. The reaction contained 100 mM Hepes (pH 7.5), 10 mM MgCl_2_, 2 mM CTP, 1 mM Kdo, 200 μM acceptor 1, and 40 μg of KrkA_GT__140_ WT or a given mutant. 5 μg *E coli* KdsB were used to initiate the reaction. Similar reagents were used for the reaction of KrkAΔC with the addition of 5 mM 5- PRPP. Reactions were quenched with an equal volume of ice-cold acetonitrile and the precipitated protein was removed by centrifuging at 21,000×*g* for 10 min before TLC or HPLC analysis.

### TLC

5 μl of the above reaction was spotted on an aluminum backed silica-based TLC plate (200 μM thickness F-254 indicator, SilliaPlate; Silicycle). A mobile phase consisting of chloroform, methanol, water and acetic acid in the following ratio 10:10:2:2 was used to resolve the plate. Once dried, the plates were imaged using a handheld UV lamp (short-wave mode) in a Bio-Rad Universal Hood II Gel Doc.

### HPLC analysis of KrkA reactions

Normal phase HPLC analysis on an Agilent 1260 Infinity LC system equipped with a 1260 Infinity II Variable Wavelength Detector was used for analyzing the KrkA_GT__140_ reactions. 10 μl samples were injected and separated using a GLYCOSEP N normal phase column (4.6 × 250 mm, Prozyme) with the following solvents: 10 mM ammonium formate pH 4.4 in 80% acetonitrile (A), 30 mM ammonium formate pH 4.4 in 40% acetonitrile (B) and 0.5% formic acid (C). The gradient conditions used were the same as used elsewhere (28). The column temperature was set to 30 °C and samples were detected using ultraviolet absorbance set at 260 nm. The gradient conditions were set as follows: a linear gradient of 100% A to 100% B over 160 min (0.4 ml/min), followed by 2 min gradient of 100% B to 100% C. Returning to 100% A over 2 min, holding for 15 min at 100% A (1 ml/min), followed by 0.4 ml/min for 5 min. HPLC results were analyzed using Agilent OpenLAB revision A.02.16. Unreacted acceptor and product eluted at 16 min and 26 min respectively. The acceptor and product peaks were manually integrated using OpenLAB CDC Chemstation vC.01.07. SR3 and the areas were used to calculate the ratio percentage of acceptor to product in the reaction. Data was plotted and analyzed using GraphPad Prism V8.3. Reported values are based on 3 technical replicates conducted using fresh protein from the same purification.

### LC-MS methods for protein molecular weight measurements

Liquid chromatography–mass spectrometry analyses were performed on Waters Acquity I-class HPLC coupled to a Synapt G2Si mass spectrometer at the Mass Spectrometry Facility of the Advanced Analysis Centre, University of Guelph. An Acquity UPLC Protein BEH C4 column (2.1 × 150 mm, 1.7 μm, 300 Å, Waters) was used for protein desalting with the following solvents, water with 0.1% formic acid for A and acetonitrile with 0.1% formic acid for B. The mobile phase gradient was as follows: initial conditions, 5% B for 2 min increasing to 60% B in 5 min and then to 85% B in 3 min followed by column wash at 85% B for 1 min and 5-min re-equilibration. The flow rate was maintained at 0.15 ml/min. The mass spectrometer electrospray capillary voltage was maintained at 3.0 kV, the source temperature was 80 °C, cone gas 50 L/h, desolvation temperature was 600 °C and desolvation gas flow was 1000 L/pressure with a nebulizing gas flow of 7 bar. The mass-to-charge ratio was scanned across the m/z range of 300 to 2000 m/z in MS resolution mode. The instrument was externally calibrated with the ESI TuneMix (Agilent). The sample injection volume was 4 μl. Data analysis was performed using the MassLynx Version 4.2 software (Waters) using the MaxEnt 1 algorithm to deconvolute the raw data.

### UPLC-MS-MS analysis of reaction products

Liquid chromatography–mass spectrometry analyses were performed on Waters Acquity I-class HPLC coupled to a Synapt G2Si mass spectrometer at the Mass Spectrometry Facility of the Advanced Analysis Centre, University of Guelph. A C18 column (Agilent Poroshell 120, 50 mm × 4.6 mm 2.7 μm) was used for chromatographic separation with the following solvents, water with 15 mM of ammonium formate (A) and acetonitrile (B). The mobile phase gradient was as follows: initial conditions were 2% B held for 2 min then increasing to 98% B in 18 min followed by column wash at 98% B for 1 min and 10 min re-equilibration. The flow rate was maintained at 0.4 ml/min. The mass spectrometer electrospray capillary voltage was maintained at 2.5 kV, the source temperature was 120 °C, cone gas 10 L/h, desolvation temperature was 300 °C and desolvation gas flow was 1000 L/h with a nebulizing gas flow of 6.5 bar. The mass-to-charge ratio in negative ion detection mode was scanned across the m/z range of 100 to 2000 m/z in MS resolution mode using Tof-MS mode. The instrument was externally calibrated with sodium iodide. The sample injection volume was 1 μl. Data analysis was performed using the MassLynx Version 4.2 software (Waters).

### Digestion and MS analysis of the kdo labelled peptide

The protein solution was denatured in 6 M urea/2 M thiourea (in 10 mM Hepes, pH 8.0), reduced in 10 mM dithiothritol (in a 50 mM ammonium bicarbonate [ABC] buffer), and alkylated in 55 mM iodoacetamide (in 50 mM ABC). Protein was then digested overnight with trypsin (Promega) and subsequently dried under vacuum. The Vanquish Neo UHPLC system, configured for trap and elute analysis, was coupled with Orbitrap Exploris 240 mass-spectrometer using the Easy-Spray source for nano LC-MS protein identification. Peptides were first trapped and washed on a Pepmap Neo C18 trap column (5 μm, 300 μm × 5 mm) then separated on EASY-Spray columns 75 μm I.D. × 50 cm with the maximum pressure of 1200 bar. The nanoLC-MS system was controlled with Standard Instrument Integration for Xcalibur software. All hardware and data acquisition software were from Thermo Fisher Scientific. The mobile phase A and weak wash liquid was water with 0.1% FA, and the mobile phase B and strong wash liquid was 80% acetonitrile with 0.1% FA. The gradient was as follows 4 to 45% B over 30 min with a flow rate of 300 nl/min. The autosampler temperature was 7 °C, and the column temperature was 45 °C. The sample (5 μl) was injected with Fast Loading set to 'Enabled' with Pressure Control at 500 bar. The column Fast Equilibration function was set to ‘Enabled’ with Pressure control at 800 bar, and the equilibration factor was set to 3. Vial bottom detection was set to ‘Enabled’.

The Orbitrap Exploris 240 MS was operated in data-dependent acquisition mode using a full scan with *m*/*z* range 375 to 1500, Orbitrap resolution of 60,000, normalized automatic gain control target value 300%, and maximum injection time set to Auto. The intensity threshold for precursor was set to 1 × 104. MS/MS spectra starting from 120 *m*/*z* were acquired in data-dependent acquisition mode with a cycle time of 2 s, where the precursors were isolated in a window of 1.6 Da and subsequently fragmented with high-energy collision dissociation using normalized collision energy of 30%. Orbitrap resolution was set to 15,000 for MS2. The normalized automatic gain control target was standard, and the maximum injection time was set to Auto. Raw data was analyzed using PEAKS Online 12 build 2.1.2024-11 to 20_160513 (Bioinformatics Solutions Inc, Waterloo, Ontario, Canada). PEAKS DB was set up to search the protein sequence with a parent mass tolerance of 10 ppm and fragment mass tolerance of 0.02 Da. The following variable modifications were considered: oxidation of methionine, deamidation of asparagine and glutamine. Cysteines were all considered to be carbamidomethylated or modified with Kdo. The results were filtered to a less than 1% false discovery rate. 13 peptides were obtained with the Kdo (+220.06) modification. The peptide SALFVGQTLNCK (m/z = 750.867) was chosen as the representative peptide.

### Protein crystallization and structure determination

Purified KrkA_GT__140_ was diluted to 1 to 10 mg/ml in the above-described minimal buffer and supplemented with 2 mM CMP (BioBasic). Crystallization experiments were conducted at room temperature in a sitting drop vapor diffusion configuration with the protein mixed with the well solution in either 2:1 or 1:1 ratio, and then equilibrated with 80 μl of well solution. For the CMP complex and adduct structures, the well solution was comprised of 0.2 M potassium chloride, 0.1 M glycine pH 9.5 and 20% v/v pentaerythritol ethoxylate (15/4 EO/OH); for the ternary complex, the well solution was comprised of 0.2 M ammonium chloride, 0.1 M Hepes pH 7.5 and 25% v/v glycerol ethoxylate. Crystals grew overnight to 3 days after setting screens. Crystals were cryoprotected with paratone-N oil before being frozen in liquid nitrogen. Data was collected at the Canadian Light Source Beam Line ID (CMP complex) or the National Synchrotron Light Source II in the Brookhaven National Laboratory (BNL) (adduct and ternary complexes) at 100K. Datasets were processed using the XDS package and scaled using XSCALE (29). The KrkA_GT__140_ WT structure was determined through molecular replacement in Phenix (1.20.1–4487) Phaser using the AlphaFold 3 generated model of KrkA_GT__140_ as the search model; the structure of the D193C adduct was determined using the wild-type structure as a search model, while the ternary complex structure used the D193C adduct structure as a search model (30). Autobuild was used for initial rebuilding followed by iterative cycles of rebuilding on Coot (31), and refinement in Phenix.refine (32). Data collection and structure refinement statistics are summarized in Table 2. Pymol was used to prepare structural figures. A model for CMP-β-Kdo binding was generated by manually orienting CMP-β-Kdo from a DALI aligned WbbB D232N (8csbA) onto the active site of KrkA_GT__140_ WT structure.

## Data availability

Atomic models have been deposited in the Protein Databank with PDB IDs 9YFX (WT CMP complex) 9YFY (Kdo adduct) and 9YFZ (ternary complex).

## Supporting information

This article contains supporting information (33).

## Conflict of interest

The authors declare that they have no conflicts of interest with the contents of this article. Matthew S. Kimber is an Editorial Board Member for this journal and was not involved in the editorial review or the decision to publish this article.
