# Supplementary material for: The retaining kdo transferase that synthesizes Escherichia coli K13 capsule is deeply divergent from structurally homologous enzymes
Source: J Biol Chem. 2026 Jan 13;302(3):111162. doi: 10.1016/j.jbc.2026.111162 (PMC12887879; doi:10.1016/j.jbc.2026.111162)
Supplement: Supporting Information [file mmc1.docx]

**Supplementary tables and figures**

| **#** | **b** | **b-H2O** | **b-NH3** | **b(2+)** | **Seq** | **y** | **y-H2O** | **y-NH3** | **y(2+)** | **#** |
| --- | --- | --- | --- | --- | --- | --- | --- | --- | --- | --- |
| **1** | 88.04 | 70.03 | 71.01 | 44.52 | S |  |  |  |  | 12 |
| **2** | 159.08 | 141.07 | 142.05 | 80.04 | A | 1413.69 | 1395.68 | 1396.67 | 707.35 | 11 |
| **3** | 272.16 | 254.15 | 255.13 | 136.58 | L | 1342.66 | 1324.65 | 1325.63 | 671.83 | 10 |
| **4** | 419.23 | 401.22 | 402.22 | 210.11 | F | 1229.56 | 1211.56 | 1212.54 | 615.29 | 9 |
| **5** | 518.3 | 500.29 | 501.27 | 259.65 | V | 1082.51 | 1064.49 | 1065.48 | 541.75 | 8 |
| **6** | 575.32 | 557.31 | 558.29 | 288.16 | G | 983.44 | 965.42 | 966.41 | 492.22 | 7 |
| **7** | 703.38 | 685.37 | 686.35 | 352.19 | Q | 926.41 | 908.4 | 909.39 | 463.71 | 6 |
| **8** | 804.44 | 786.42 | 787.4 | 402.71 | T | 798.35 | 780.34 | 781.33 | 399.68 | 5 |
| **9** | 917.51 | 899.5 | 900.48 | 459.25 | L | 697.31 | 679.3 | 680.28 | 349.15 | 4 |
| **10** | 1031.55 | 1013.54 | 1014.53 | 516.28 | N | 584.22 | 566.21 | 567.2 | 292.61 | 3 |
| **11** | 1354.62 | 1336.61 | 1337.59 | 677.81 | C(+220.06) | 470.18 | 452.17 | 453.15 | 235.59 | 2 |
| **12** |  |  |  |  | K | 147.11 | 129.1 | 130.09 | 74.06 | 1 |

Table S1. MS-MS analysis of the KrkA tryptic digestion peptide with mass 1499.7174

| **Search model** | **PDB** | **protein** | **Z-score** | **r.m.s.d. (Å)** | **aligned** |
| --- | --- | --- | --- | --- | --- |
| KrkA_GT140_ | 8csd | WbbB | 12.9 | 3.8 | 241 |
| KrkA_GT140__N | 8csd | WbbB | 4.3 | 3.5 | 138 |
| KrkA_GT140__C | 8csd | WbbB | 10.2 | 3.3 | 112 |
| KrkA_GT140_ | 6mgd | KpsC | 10.6 | 3.6 | 206 |
| KrkA_GT140__N | 6mgd | KpsC | 3.5 | 3.5 | 86 |
| KrkA_GT140__C | 6mgd | KpsC | 11.4 | 2.3 | 110 |
| KrkA_GT140_ | 5wcn | SiaD | 10.2 | 4.8 | 168 |
| KrkA_GT140__N | 5wcn | SiaD | 2.5 |  |  |
| KrkA_GT140__C | 5wcn | SiaD | 10.8 | 3.1 | 124 |

Table S2. DALI superposition statistics between KrkA and related proteins. The KrkA_GT140_ _N model encompasses residues 1 to 154 and 295 to 351, the KrkA_GT140_ _C model residues 155 to 294.

| **protein** | **SiaD** | **KrkA_GT140_** | **WbbB** | **KpsC** | **KpsS1** |
| --- | --- | --- | --- | --- | --- |
| **source** | 5wcnA | this work | 8csdA | 6mgdA | AF3 |
| **5wcnA** | 61.4 | 10.2 | 6.4 | 9 | 10.9 |
| **KrkA_GT140_** | 10.2 | 57.4 | 13 | 11.3 | 12.5 |
| **8csdA** | 6.4 | 13 | 69.7 | 14.8 | 16.9 |
| **6mgdA** | 9 | 11.3 | 14.8 | 49.3 | 17.2 |
| **KpsS1** | 10.9 | 12.5 | 16.9 | 17.2 | 63 |

Table S3. All vs all DALI Z-score comparison of KrkA_GT140_ and various homologs. The structure with source “AF3” is an Alphafold 3 model.

| Variant | Primers/Construct |
| --- | --- |
| KrkA | Synthesized by Twist Bioscience |
| KrkAΔC | **Forward Primer (5’to 3’)**  GATCGACACCACCACTGAGATCCGGCTAACAAA  **Reverse Primer (3’ to 5’)**  TCGATCTTAATTAATAAATAACGATGCGGCTTCTTTCCAG |
| KrkA_GT140_ | Synthesized by Twist Bioscience |
| E17A | Synthesized by Twist Bioscience |
| N21A | Synthesized by Twist Bioscience |
| K71A | **Forward Primer (5’to 3’)**  GAGGATGCGCAATTCTATTACAATGAGAGGGAT  **Reverse Primer (3’ to 5’)**  GAATTGCGCATCCTCAACATACTCTATATTTGA |
| E102A | **Forward Primer (5’to 3’)**  GTGGGGTAT**GCG**TTATCAGAGCAAACAAGAAAAATTCTC  **Reverse Primer (3’ to 5’)**  CTCTGATAACGCATACCCCACAAGAATAATGTCATTTTT |
| E102Q | **Forward Primer (5’to 3’)**  GTGGGGTAT**CAG**TTATCAGAGCAAACAAGAAAAATTCTC  **Reverse Primer (3’ to 5’)**  CTCTGATAACTGATACCCCACAAGAATAATGTCATTTTT |
| R127A | Synthesized by Twist Bioscience |
| R170A | Synthesized by Twist Bioscience |
| D193A | **Forward Primer (5’to 3’)**  ACACTAAAC**GCG**AAAGCAGTCTTCCATAATGGAAA  **Reverse Primer (3’ to 5’)**  GACTGCTTTCGCGTTTAGTGTTTGGCCCACAAACA |
| D193C | **Forward Primer (5’to 3’)**  ACACTAAAC**TGC**AAAGCAGTCTTCCATAATGGAAA  **Reverse Primer (3’ to 5’)**  GACTGCTTTGCAGTTTAGTGTTTGGCCCACAAACA |
| D193N | **Forward Primer (5’to 3’)**  ACACTAAAC**AAC**AAAGCAGTCTTCCATAATGGAA  **Reverse Primer (3’ to 5’)**  GACTGCTTTGTTGTTTAGTGTTTGGCCCACAAACA |
| H227A | **Forward Primer (5’to 3’)**  TATAGCCGA**GCG**CCATTTGTTAAAGATGGAGATGAA  **Reverse Primer (3’ to 5’)**  AACAAATGGCGCTCGGCTATAATAAACATGATTGTA |

Table S4. Details of constructs. Variants with listed primers were amplified using the listed primers. Other constructs were ordered from Twist, as noted.


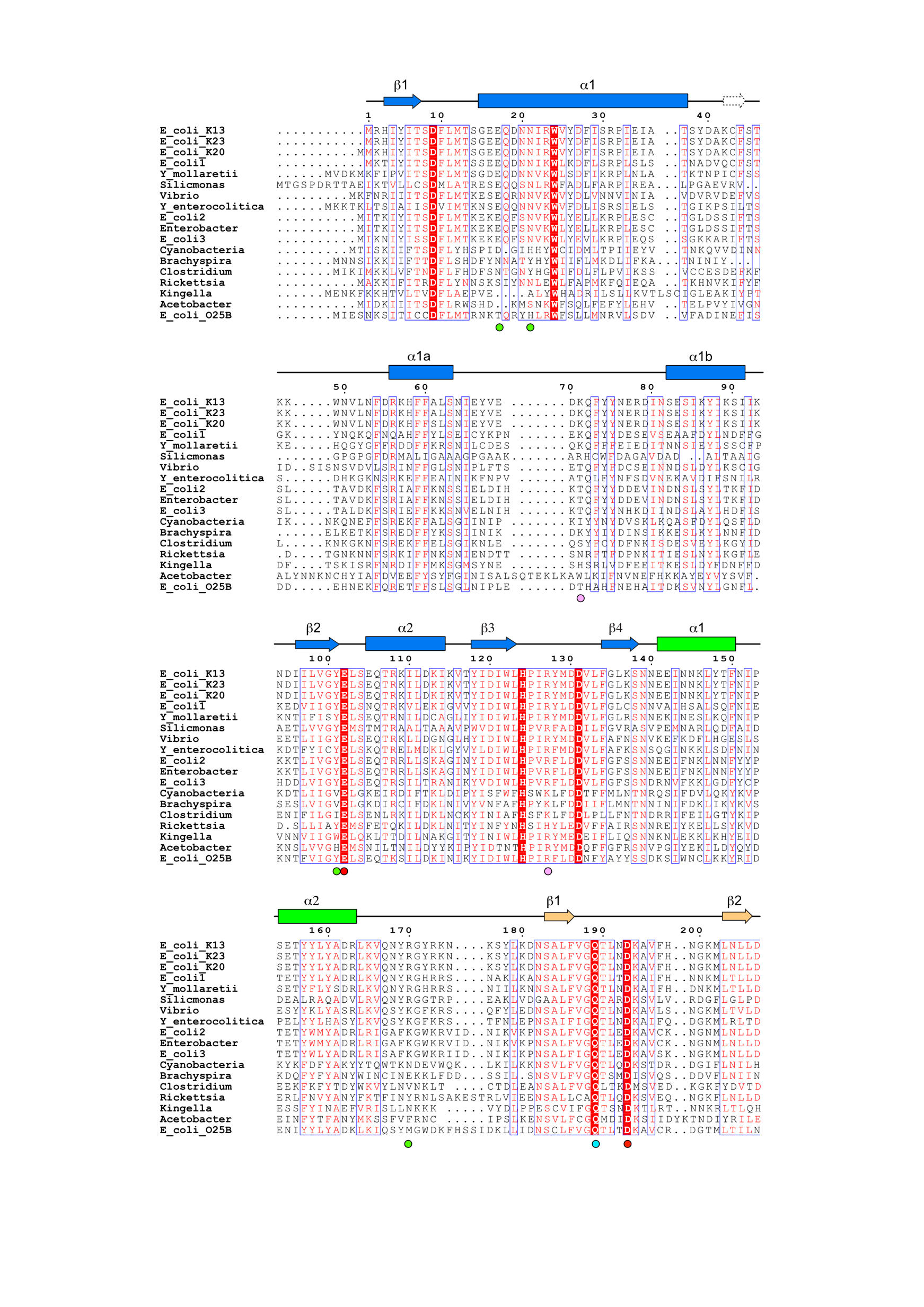

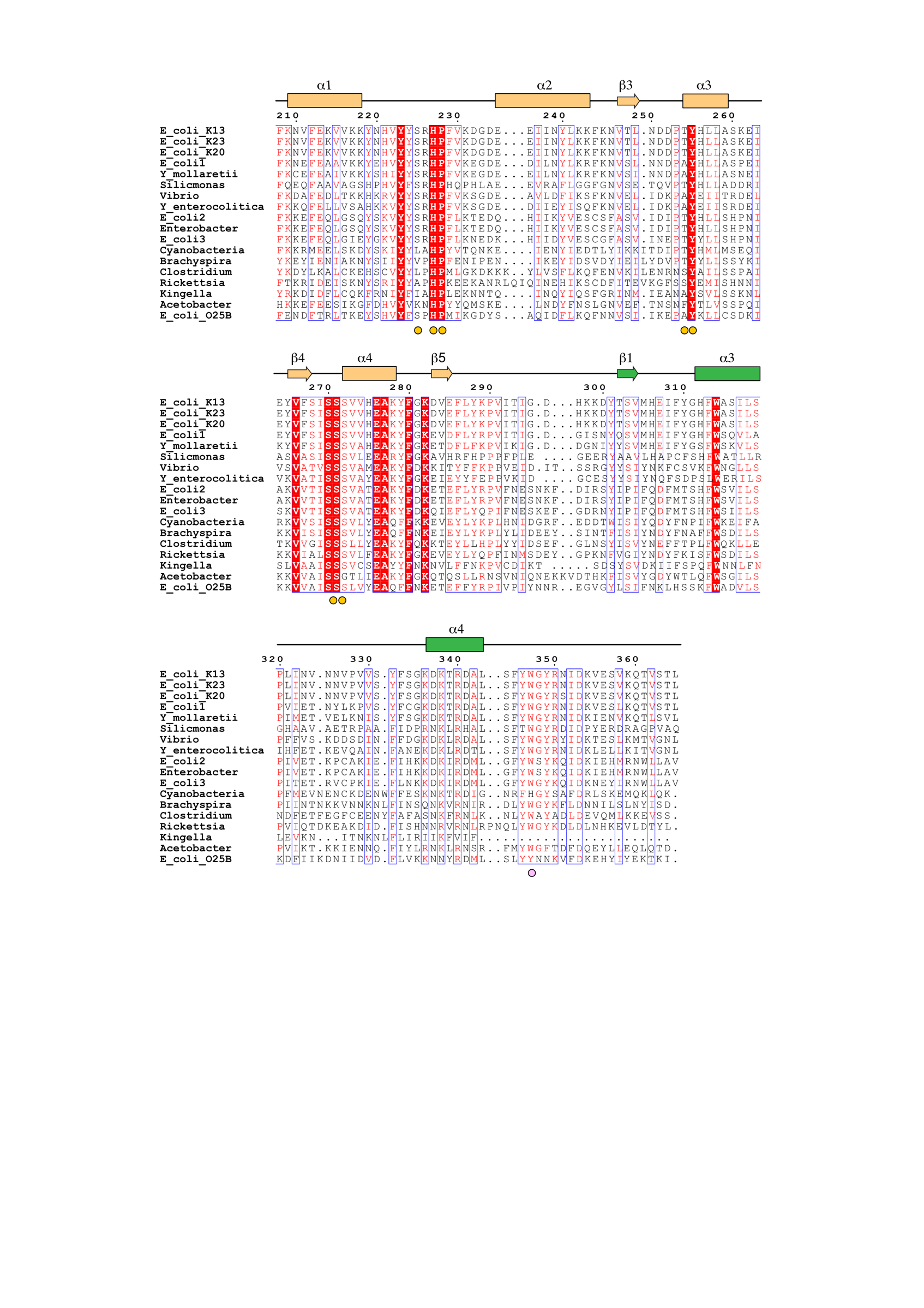


Figure S1. Multiple sequence alignments of KrkA sequence homologs, with secondary structure for KrkA as shown in Figure 4C depicted above the sequences. The displayed sequence only covers the GT140 module. Red circles denote catalytic residues, orange circles denote CMP binding residues, pink circles denote Kdo adduct binding residues, and green circles denote acceptor binding residues. The corresponding accession codes are:

E_coli_K13 VED05356.1, E_coli_K23 VEC21054.1, E_coli_K20 AUT31333.1, E_coli1 HBD5655969.1, Y_mollaretii WP_271297553.1, Silicmonas MCG6883119.1, Vibrio WP_315610799.1, Y_enterocolitica HHH0502579.1, E_coli2 EFK3614542.1, Enterobacter WP_434369629.1, E_coli3 HEL7985316.1, Cyanobacteria MBE7703456.1, Brachyspira WP_157159463.1, Clostridium MBS4759537.1, Rickettsia MDC0857264.1, Kingella WP_003788872.1, Acetobacter MBS5834713.1, E_coli_O25B HAI7522068.1.


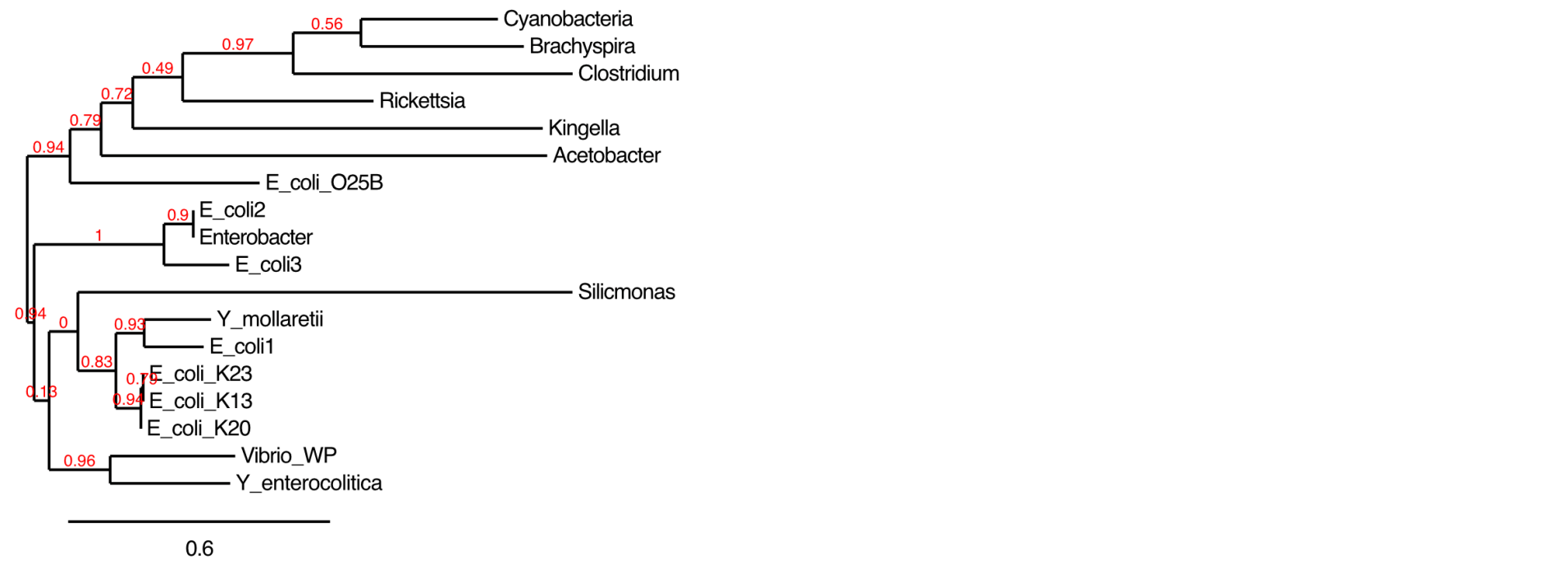


Figure S2. Phylogenetic tree of KrkA_GT140_ homologs. Sequence names as in the caption to figure S1.


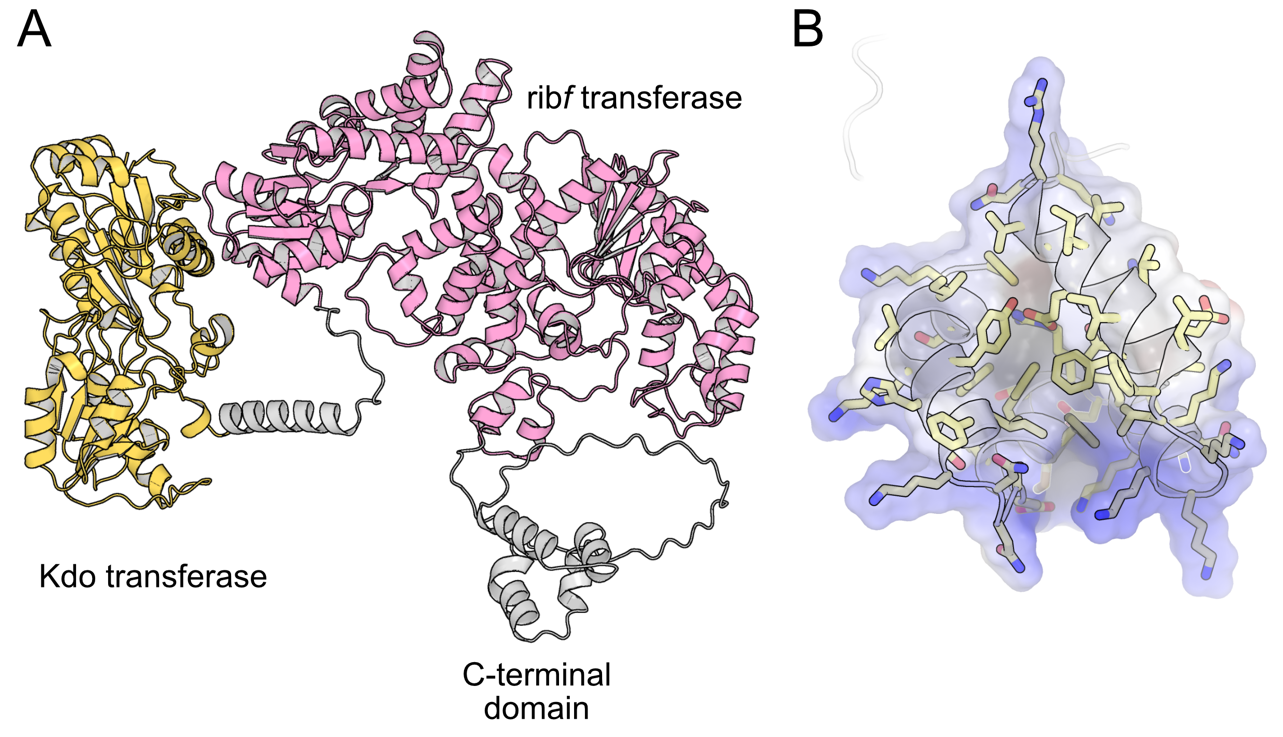


Figure S3: Alphafold 3 model of full-length KrkA. A) A cartoon trace, emphasizing the modular organization of KrkA. B) Details of the C-terminal domain, shown as cartoon, sticks and a semi-transparent electrostatic surface. This module is highly electropositive and exposes multiple non-polar residues along its surface, suggesting that it may mediate membrane interactions.


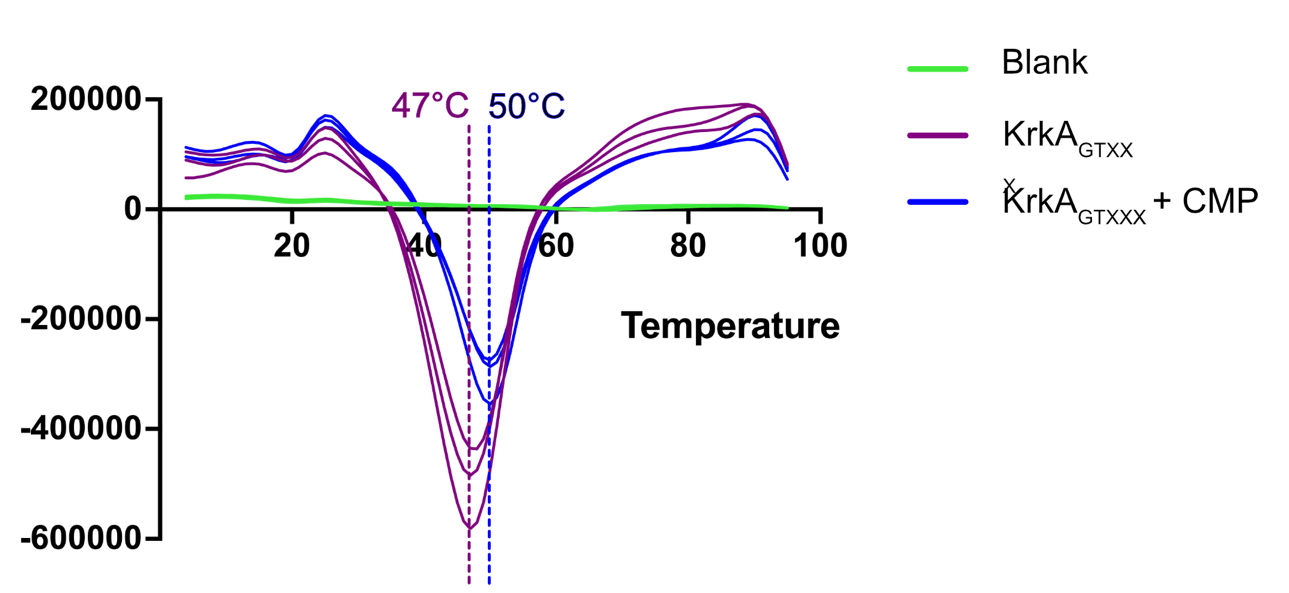


Figure S4. Differential scanning fluorimetry (DSF) melt curves for KrkA_GT140_ wild type

in the absence and presence of 2 mM CMP.


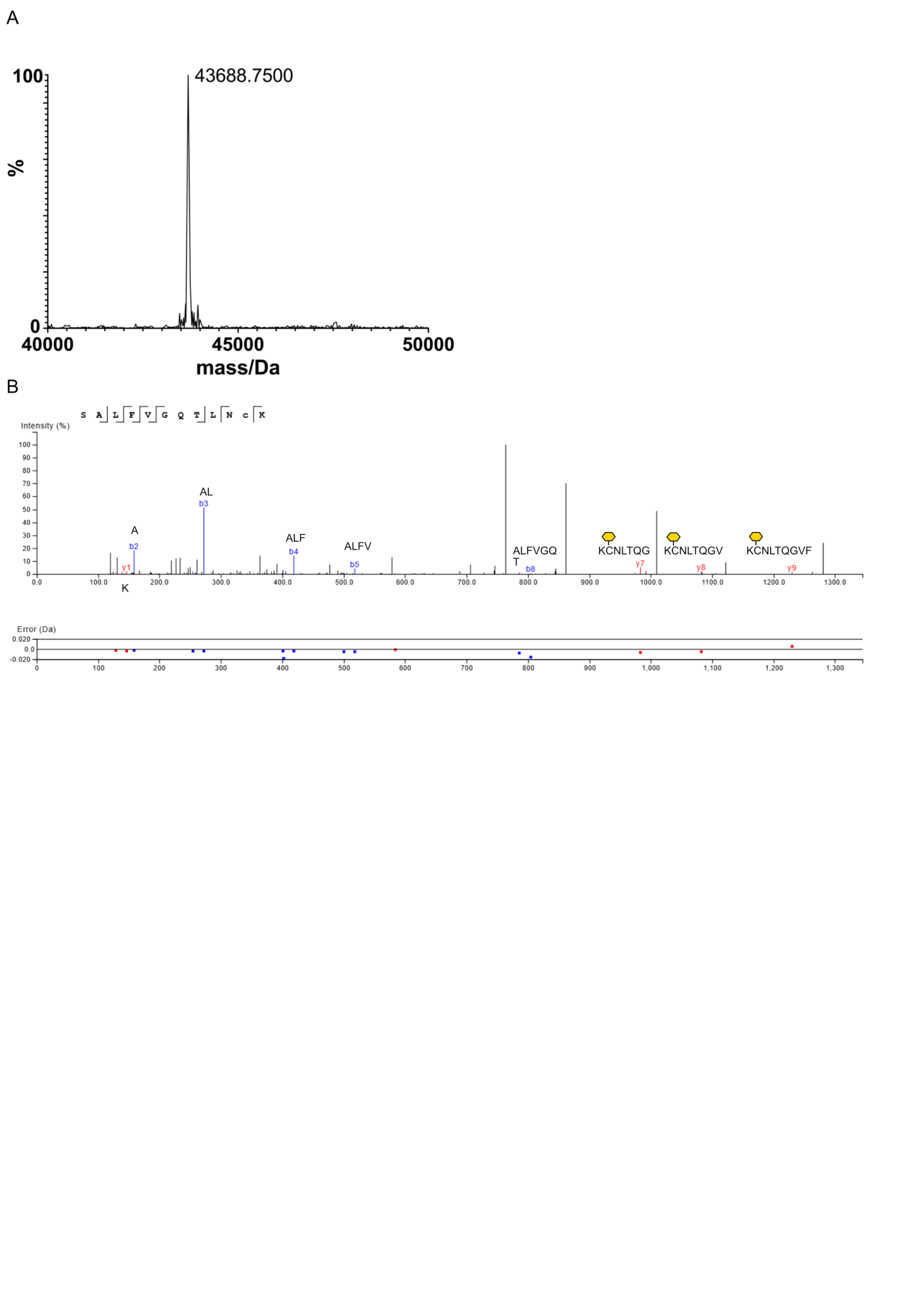


Figure S5. Mass spectrometric analysis of the KrkA_GT140_ D193C variant as expressed in *E. coli*. A) MS analysis of the intact protein. The peak at 43688.75 Da is 220 Daltons heavier than the expected mass for this construct, suggesting that the protein has been modified stoichiometrically by a single Kdo residue. B) The upper panel sows MS fragmentation analysis of the KrkA _GT140_ D193C tryptic digest peptide SALFVGQTLNCK (m/z = 750.867). The y7, y8 and y9 fragments show the addition of a 220.06 Da mass, consistent with the addition of a single Kdo residue. The lower panel reports error from the expected mass for each of the labelled b and y peptides.


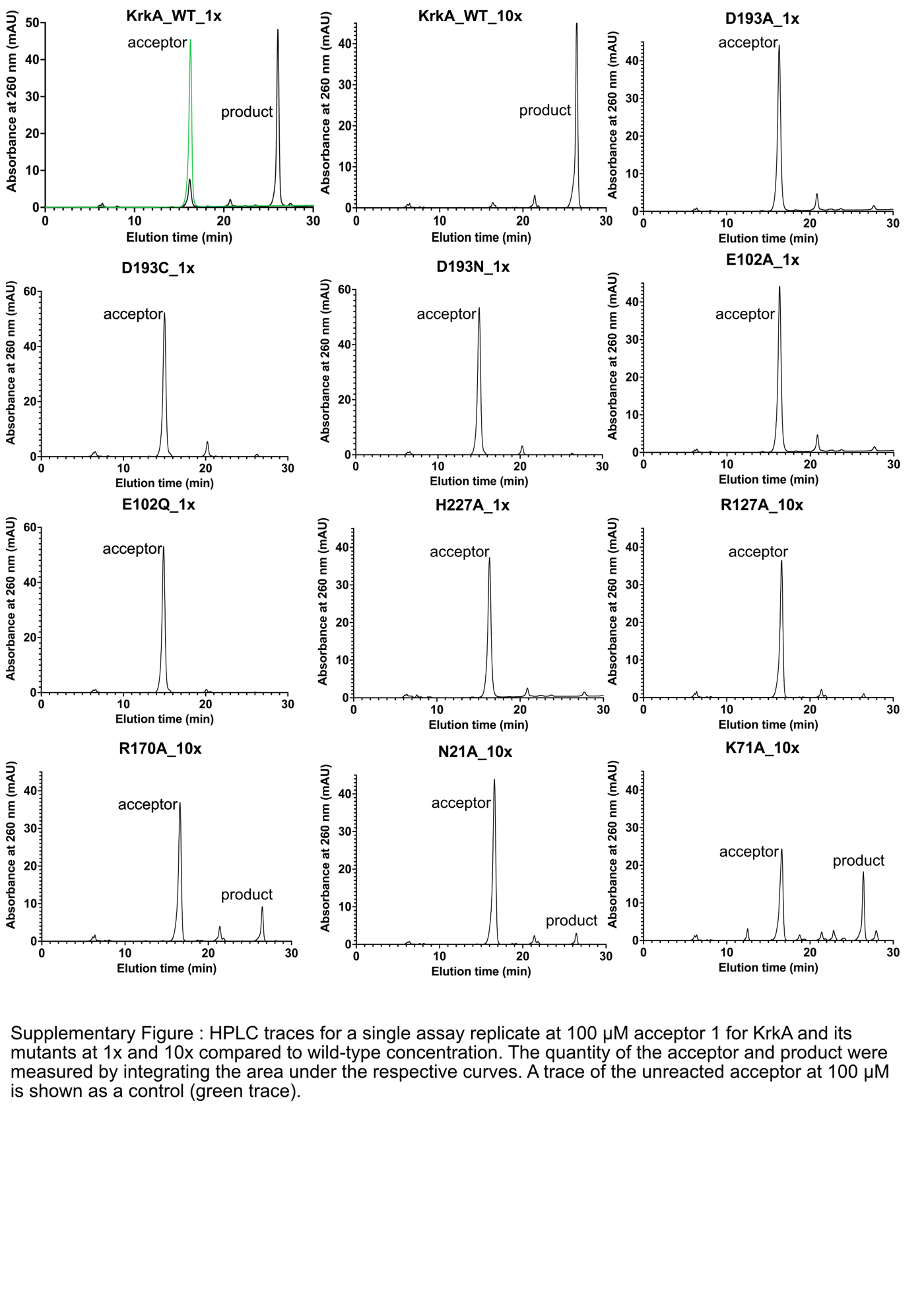


Figure S6: HPLC traces for a single assay replicate at 100 µM acceptor 1 for KrkA and its variants at 1x and 10x the reference wild-type concentration. The quantity of the acceptor and product were measured by integrating the area under the respective curves. A trace of the unreacted acceptor at 100 µM is shown as a control (green trace).


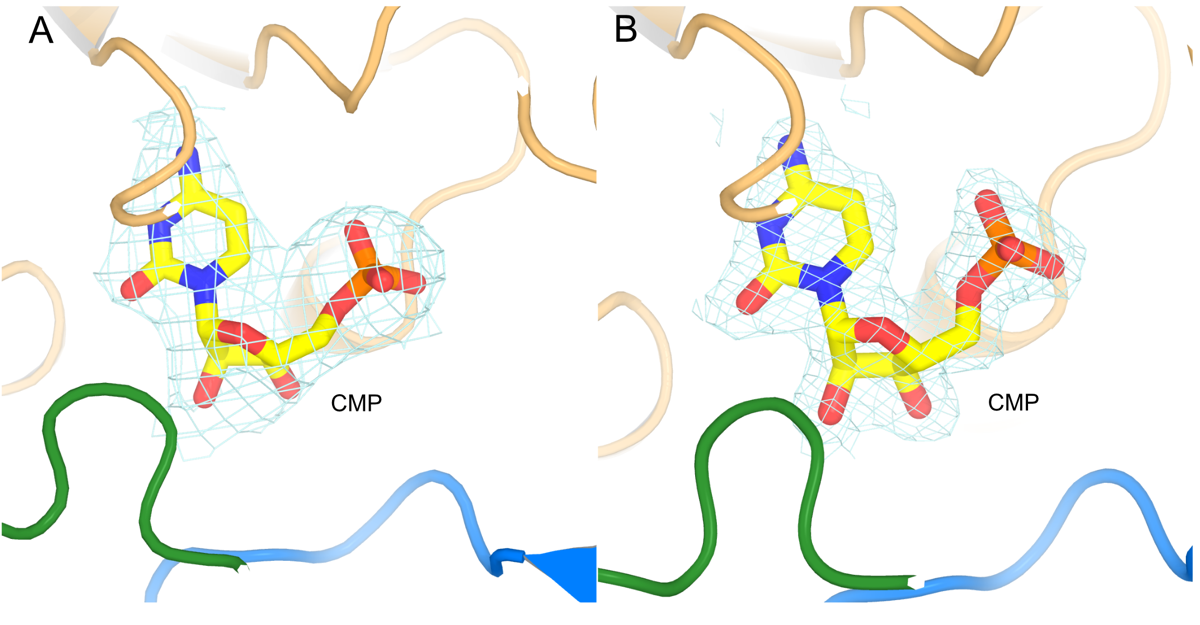


Figure S7. Electron density for the CMP substituent. A) 2F_o_-F_c_ electron density in the wild-type structure at 2.7 Å resolution, contoured at 1.0 σ. B) The equivalent 2F_o_-F_c_ electron density contoured around CMP in the D193C ternary complex structure, at 2.0 Å resolution, contoured at 1.0 σ.


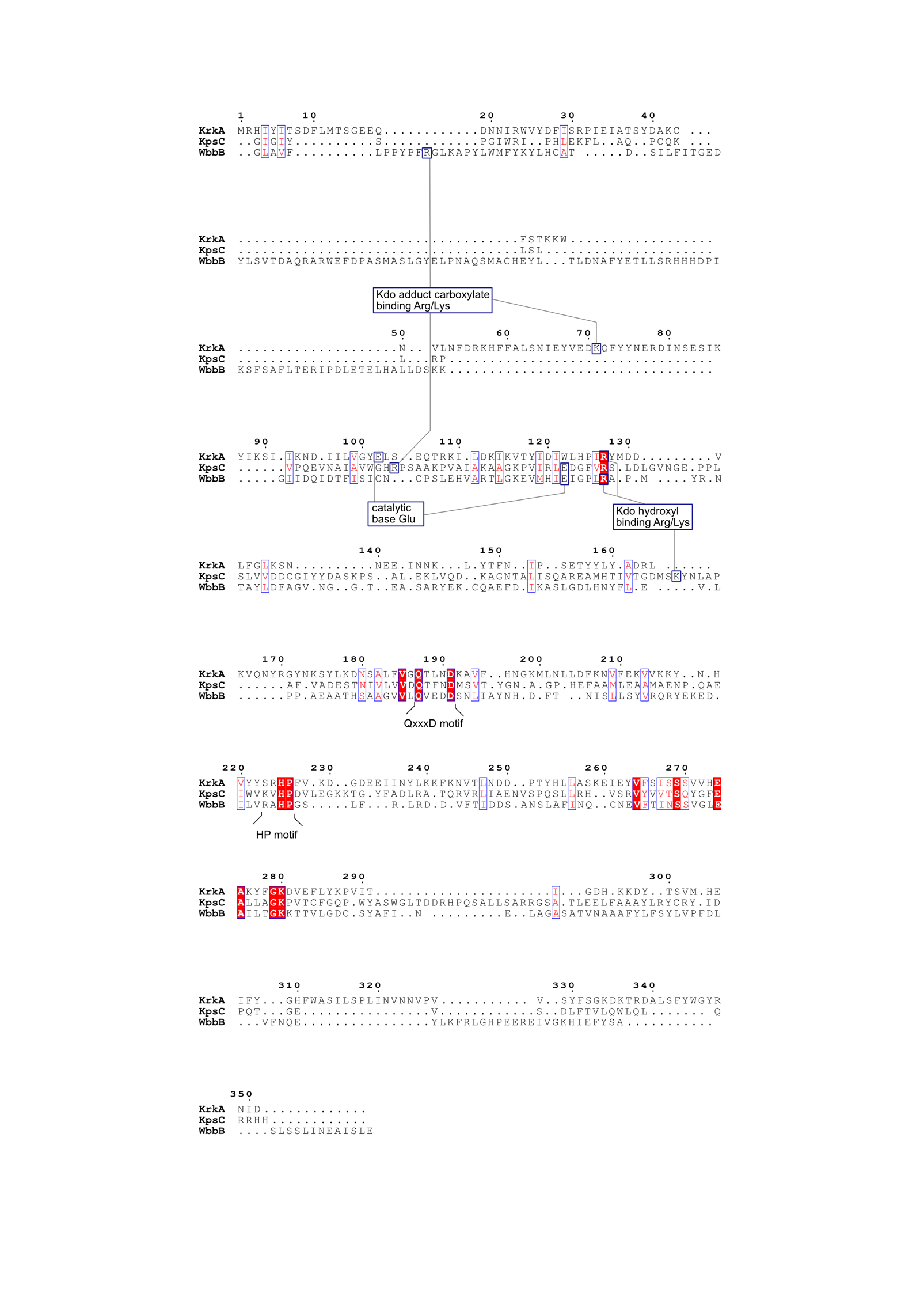


Figure S8. Structure-based sequence alignment of KrkA, KpsC and WbbB. Note that several key groups, including the catalytic base, are contributed by topologically distinct residues.


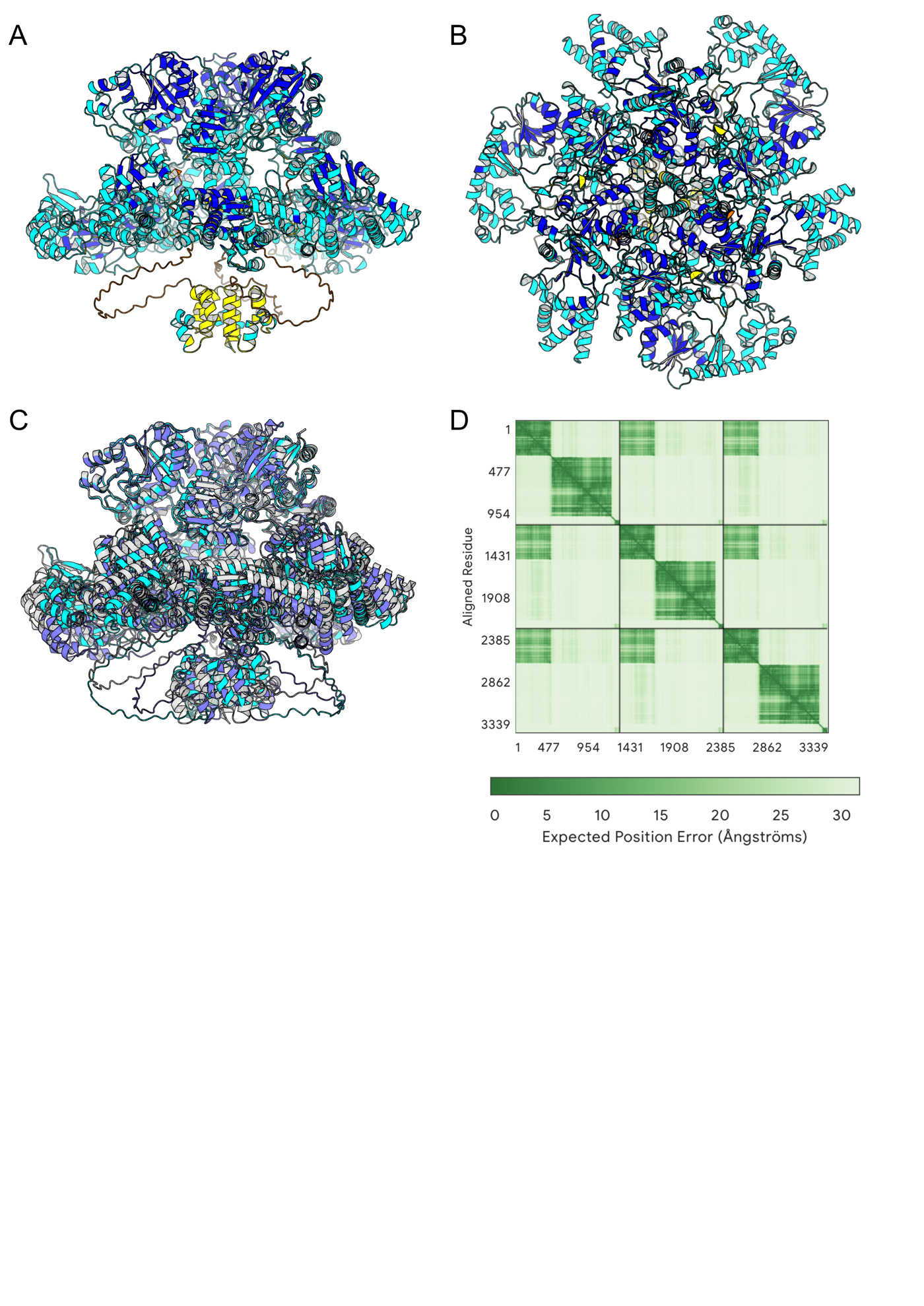


Figure S9. Alphafold 3 prediction of the full-length KrkA trimer. A, B) orthogonal views of the KrkA trimer, colored by pLDDT. Blue denotes pLDDT scores greater than 90, cyan pLDDT scores between 70 and 90, yellow pLDDT scores between 50 and 70, and orange, pLDDT scores less than 50. C) Superposition of three different AF3 predictions, superimposed on the GT140 domain (topmost) of one of them. D) Predicted alignment error plotted for one AF3 prediction. While each module is individually predicted reliably, and the GT140 modules are reliably placed relative to one another, the relative positions of the ribosyltransferase modules relative to the GT140 domains or one another is poorly defined, suggesting that they may be mobile.


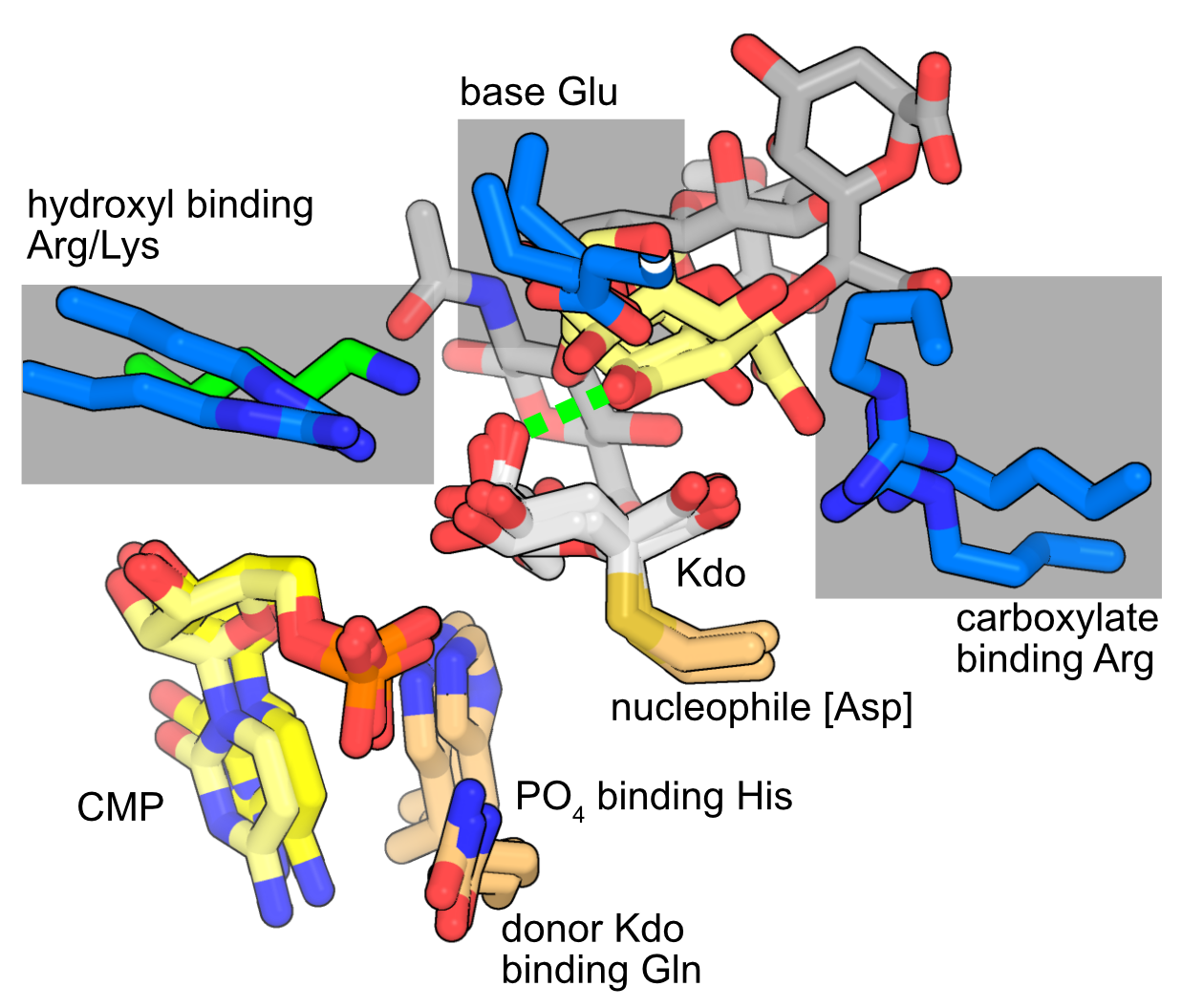


Figure S10. Conserved and convergent elements in retaining Kdo-transferase structures. The figure shows key residues from manually superimposed ternary complex structures of KrkA_GT140_, WbbB_GT99_ and KpsC_N_. The phosphate binding histidine, donor carboxylate binding glutamine and nucleophilic aspartate contributed from the C-terminal domain are all conserved, while the basic residues that co-ordinate the carboxylate group and hydroxyl groups of the adduct, as well as the catalytic glutamate base, converge upon similar positions from topologically different points in the N-terminal domain.

**Figure S11.** Synthesis of acceptor **1**.

**Methyl 8-amino-1-octyl 2,3,5-tri-*O*-benzoyl-β-D-ribofuranosyl-(1→7)-8-*O*-tert-butyldiphenylsilyl-4,5-*O*-isopropylidene-3-deoxy-β-D-*manno*-2-octulopyranosidonate (4).** To a stirred solution of acceptor **2** (1) (120.0 mg, 0.176 mmol) and donor **3** (120.0 mg, 0.211 mmol) in dry CH_2_Cl_2_ (5 mL) was added 4 Å molecular sieves powder (500 mg). After stirring for 30 min at room temperature, the reaction mixture was cooled to 0 °C and then *N*-iodosuccinimide (79.2 mg, 0.352 mmol) and silver trifluoromethanesulfonate (9.0 mg, 0.0352 mmol) were added successively. The resulting solution was warmed to room temperature and stirred for 1 h. Triethylamine was added to the mixture and the solution was diluted with CH_2_Cl_2_ and then filtered through Celite. The filtrate was then washed with saturated aqueous Na_2_S_2_O_3_ and saturated aqueous NaHCO_3_. The organic layer was dried over Na_2_SO_4_, filtered and concentrated to dryness. The crude residue was purified by flash chromatography (3:1 hexanes–EtOAc) to afford **4** (155 mg, 78%) as a white solid: *R_f_* = 0.43 (3:1 hexanes–EtOAc); [α]_D_^25^ –28.2 (*c* = 0.39, CHCl_3_); ^1^H NMR (600 MHz, CDCl_3_) δ 8.03–7.97 (m, 4H, Ar), 7.87–7.82 (m, 2H, Ar), 7.72–7.65 (m, 4H, Ar), 7.62–7.57 (m, 1H, Ar), 7.51–7.46 (m, 2H, Ar), 7.47–7.41 (m, 2H, Ar), 7.39–7.22 (m, 10H, Ar), 5.87 (app td, *J* = 4.7, 1.7 Hz, 1H, H-3’), 5.77 (d, *J* = 4.5 Hz, 1H, H-2’), 5.69 (s, 1H, H-1’), 4.81–4.55 (m, 3H, H-5a’, H-5b’, H-4’), 4.36 (dd, *J* = 6.8, 2.0 Hz, 1H, H-5), 4.31 (app dt, *J* = 7.0, 5.2 Hz, 1H, H-4), 4.26 (ddd, *J* = 8.4, 6.1, 1.9 Hz, 1H, H-7), 4.15 (dd, *J* = 11.8, 1.9 Hz, 1H, H-8a), 3.94 (dd, *J* = 11.9, 6.1 Hz, 1H, H-8b), 3.73 (dd, *J* = 8.7, 2.0 Hz, 1H, H-6), 3.61 (s, 3H, COOC*H*_3_), 3.49 (app dt, *J* = 9.3, 6.6 Hz, 1H, octyl OC*H*_2_), 3.24 (app t, *J* = 7.0 Hz, 2H, octyl C*H*_2_N_3_), 3.19 (app dt, *J* = 9.3, 6.8 Hz, 1H, octyl OC*H*_2_), 2.04 (dd, *J* = 14.8, 5.5 Hz, 1H, H-3a), 1.99 (dd, *J* = 14.8, 4.8 Hz, 1H, H-3b), 1.60–1.54 (m, 2H, NCH_2_C*H*_2_), 1.50 (s, 3H, C(C*H*_3_)_2_), 1.49–1.44 (m, 2H, OCH_2_C*H*_2_), 1.37 (s, 3H, O_2_C(C*H*_3_)_2_), 1.36–1.21 (m, 8H, 4 x CH_2_), 1.04 (s, 9H, C(C*H*_3_)_3_); ^13^C NMR (151 MHz, CDCl_3_) δ 170.0 (C-1), 166.3 (C=O), 165.4 (C=O), 165.2 (C=O), 135.9 (2 x Ar), 135.7 (2 x Ar), 133.7 (Ar), 133.5 (Ar), 133.4 (Ar), 133.3 (Ar), 133.2 (Ar), 130.0 (2 x Ar), 129.9 (2 x Ar), 129.8 (Ar), 129.8 (2 x Ar), 129.8 (Ar), 129.7 (Ar), 129.5 (Ar), 129.2 (Ar), 128.6 (2 x Ar), 128.4 (2 x Ar), 128.4 (2 x Ar), 127.7 (4 x Ar), 109.3 (O_2_*C*(CH_3_)_2_), 104.2 (C-1’), 98.7 (C-2), 78.3 (C-4’), 76.0 (C-7), 76.0 (C-2’), 72.9 (C-3’), 71.1 (C-6), 70.6 (C-5), 70.4 (C-4), 65.9 (C-5’), 64.3 (octyl O*C*H_2_), 63.7 (C-8), 52.2 (COO*C*H_3_), 51.6 (octyl *C*H_2_N_3_), 33.9 (C-3), 29.8 (CH_2_), 29.4 (CH_2_), 29.2 (CH_2_), 29.0 (CH_2_), 27.1 (O_2_C(*C*H_3_)_2_), 26.9 (3 x C(*C*H_3_)_3_), 26.8 (CH_2_), 26.1 (CH_2_), 25.5 (O_2_C(*C*H_3_)_2_), 19.3 (*C*(CH_3_)_3_); HRMS (ESI–TOF) *m/z* [M + NH_4_]^+^ calcd for C_62_H_73_N_4_O_15_Si^+^ 1145.5149, found 1145.5145;

**8-Azido-1-octyl β-D-ribofuranosyl-(1→7)-3-deoxy-β-D-*manno*-2-octulopyranosidonic acid (5).** Compound **4** (135 mg, 0.120 mmol) was stirred in 1N HCl (2.0 mL) in CH_3_OH (8 mL) at 60 °C overnight. The reaction mixture was allowed to cool to room temperature then concentrated. The resulting crude product was then dissolved in EtOH–H_2_O (15 mL, 2:1) followed by addition of an aq. 1N NaOH (5.0 mL) at 50 °C. After stirring overnight, the reaction mixture was neutralized by the addition of Amberlite® IR-120 (H^+^) cation exchange resin, filtered and concentrated to dryness. The crude residue was purified by reversed phase column chromatography (0% to 40% gradient of CH_3_OH in H_2_O) using a C18 Sep-Pak cartridge column to give a product that was redissolved in distilled water. The resulting solution was frozen and then lyophilized to afford **5** (53.2 mg, 85%) as a white solid: [α]_D_^25^ +117.8 (*c* = 0.69, H_2_O); ^1^H NMR (600 MHz, D_2_O) δ 5.23 (s, 1H, H-1’), 4.22 (dd, *J* = 7.3, 4.6 Hz, 1H, H-3’), 4.12 (d, *J* = 4.6 Hz, 1H, H-2’), 4.08–4.01 (m, 2H, H-6, H-4’), 3.97–3.88 (m, 3H, H-5, H-8a, H-8b), 3.86 (dd, *J* = 12.4, 3.0 Hz, 1H, H-5a’), 3.79–3.73 (m, 1H, H-4), 3.73–3.69 (m, 2H, octyl OC*H*_2_, H-5b’), 3.68 (d, *J* = 9.3 Hz, 1H, H-7), 3.44 (app dt, *J* = 9.1, 6.8 Hz, 1H, octyl OC*H*_2_), 3.33 (app t, *J* = 6.9 Hz, 2H, octyl C*H*_2_N_3_), 2.43 (dd, *J* = 12.2, 4.7 Hz, 1H, H-3a), 1.80 (app t, *J* = 12.4 Hz, 1H, H-3b), 1.66–1.59 (m, 2H, NCH_2_C*H*_2_), 1.58–1.52 (m, 2H, OCH_2_C*H*_2_), 1.43–1.27 (m, 8H, 4 x CH_2_); ^13^C NMR (151 MHz, D_2_O) δ 173.8 (C-1), 104.8 (C-1’), 101.1 (C-2), 82.6 (C-6), 74.8 (C-2’), 74.4 (C-4’), 71.9 (C-7), 70.3 (C-3’), 67.4 (C-4), 65.1 (C-5), 64.9 (octyl O*C*H_2_), 62.3 (C-5’), 59.9 (C-8), 51.3 (octyl *C*H_2_N_3_), 34.8 (C-3), 28.9 (CH_2_), 28.3 (CH_2_), 28.1 (CH_2_), 27.9 (CH_2_), 25.9 (CH_2_), 25.0 (CH_2_); HRMS (ESI–TOF) *m/z* [M + Na]^+^ calcd for C_21_H_73_N_3_NaO_12_^+^ 546.2269, found 546.2278.

**8-(*p*-Methoxy)benzamido-1-octyl β-D-ribofuranosyl-(1→7)-3-deoxy-β-D-*manno*-2-octulopyranosidonic acid (1).** To a solution of **4** (13.0 mg, 0.0249 mmol) in THF–H_2_O (5 mL, 4:1) was added triphenylphosphine (13.0 mg, 0.0497 mmol) and 6N NaOH (1.0 µL). The solution was stirred overnight before the solvent was evaporated. The resulting crude mixture was dissolved in H_2_O (2 mL) followed by the addition of 4-methoxybenzoyl chloride (4.3 µL, 0.030 mmol) and triethylamine (10.4 µL, 0.0747 mmol). After 3 h of stirring, the mixture was concentrated to dryness. The crude residue was purified by reversed phase column chromatography (0% to 40% gradient of CH_3_OH in H_2_O) using a C18 Sep-Pak cartridge column to give a product that was redissolved in distilled water. The resulting solution was frozen and then lyophilized to afford **1** (14.2 mg, 90%) as a white solid: [α]_D_^25^ –24.2 (*c* = 0.20, H_2_O); ^1^H NMR (600 MHz, D_2_O) δ 7.79–7.70 (m, 2H, Ar), 7.10–7.03 (m, 2H, Ar), 5.19 (s, 1H, H-1’), 4.20 (dd, *J* = 7.3, 4.6 Hz, 1H, H-3’), 4.10 (d, *J* = 4.6 Hz, 1H, H-2’), 4.05–3.99 (m, 3H, H-2’, H-6, H-4’), 3.92 (d, *J* = 2.7 Hz, 1H, H-5), 3.89 (s, 3H, OCH_3_), 3.88 (dd, *J* = 13.0, 2.5 Hz, 1H, H-8a), 3.85 (dd, *J* = 12.8, 3.1 Hz, 1H, H-8b), 3.85 (d, *J* = 12.5 Hz, 1H, H-5a’), 3.74–3.66 (m, 3H, H-4, octyl OC*H*_2_, H-5b’, H-8b), 3.66 (d, *J* = 9.3 Hz, 1H, H-7), 3.41 (app dt, *J* = 9.2, 6.8 Hz, 1H, octyl OC*H*_2_), 3.37 (app t, *J* = 6.9 Hz, 1H, octyl C*H*_2_N_3_), 2.42 (dd, *J* = 12.2, 4.7 Hz, 1H, H-3a), 1.78 (app t, *J* = 12.4 Hz, 1H, H-3b), 1.65–1.58 (m, 2H, NCH_2_C*H*_2_), 1.57–1.49 (m, 2H, OCH_2_C*H*_2_), 1.40–1.26 (m, 8H, 4 x CH_2_); ^13^C NMR (151 MHz, D_2_O) δ 173.9 (C-1), 170.2 (C=O), 161.8 (Ar), 160.3 (Ar), 128.9 (2 x Ar), 126.4 (Ar), 114.0 (2 x Ar), 104.73 (C-1’), 101.05 (C-2), 82.49 (C-6), 74.74 (C-2’), 74.30 (C-4’), 71.84 (C-7), 70.25 (C-3’), 67.37 (C-4), 65.03 (C-5), 64.85 (octyl O*C*H_2_), 62.25 (C-5’), 59.79 (C-8), 55.47 (OCH_3_), 39.91 (octyl *C*H_2_NH), 34.81 (C-3), 28.84 (CH_2_), 28.30 (CH_2_), 28.2 (2 x CH_2_), 25.99 (CH_2_), 24.97 (CH_2_); HRMS (ESI–TOF) *m/z* [M + Na]^+^ calcd for C_29_H_45_NNaO_14_^+^ 654.2732, found 654.2742.

**References**

1. Gao, Z., Ovchinnikova, O. G., Huang, B.-S., Liu, F., Williams, D. E., Andersen, R. J., Lowary, T. L., Whitfield, C., and Withers, S. G. (2019) High-Throughput “FP-Tag” Assay for the Identification of Glycosyltransferase Inhibitors. *J. Am. Chem. Soc.* **141**, 2201–2204
